# Recent Advances in Power-to-X Technology for the Production of Fuels and Chemicals

**DOI:** 10.3389/fchem.2019.00392

**Published:** 2019-06-05

**Authors:** Bruna Rego de Vasconcelos, Jean-Michel Lavoie

**Affiliations:** Biomass Technology Laboratory (BTL), Department of Chemical and Biotechnological Engineering, Université de Sherbrooke, Sherbrooke, QC, Canada

**Keywords:** Power-to-X, renewable electricity, chemical storage, CO_2_ electrochemical reduction, CO_2_ hydrogenation

## Abstract

Environmental issues related to greenhouse gas emissions are progressively pushing the transition toward fossil-free energy scenario, in which renewable energies such as solar and wind power will unavoidably play a key role. However, for this transition to succeed, significant issues related to renewable energy storage have to be addressed. Power-to-X (PtX) technologies have gained increased attention since they actually convert renewable electricity to chemicals and fuels that can be more easily stored and transported. H_2_ production through water electrolysis is a promising approach since it leads to the production of a sustainable fuel that can be used directly in hydrogen fuel cells or to reduce carbon dioxide (CO_2_) in chemicals and fuels compatible with the existing infrastructure for production and transportation. CO_2_ electrochemical reduction is also an interesting approach, allowing the direct conversion of CO_2_ into value-added products using renewable electricity. In this review, attention will be given to technologies for sustainable H_2_ production, focusing on water electrolysis using renewable energy as well as on its remaining challenges for large scale production and integration with other technologies. Furthermore, recent advances on PtX technologies for the production of key chemicals (formic acid, formaldehyde, methanol and methane) and fuels (gasoline, diesel and jet fuel) will also be discussed with focus on two main pathways: CO_2_ hydrogenation and CO_2_ electrochemical reduction.

## Introduction

After COP21, most countries on the planet decided to commit to act on their Green House Gases (GHG) emissions in order to cope for the possible threats that could represent climate changes. From all nations around the world, a small handful decided not to pledge specific reductions or control over their emissions up to 2030^1^. However, reducing GHG (Table 1) may represent a far more ambiguous challenge than it seems because of the significant consumption of energy that is being made. In addition, such tendency is actually increasing proportionally with the portion of the globe's population reaching for the middle class (Dulal et al., 2011).

**Table 1:** List of acronyms.

GHG	Greenhouse gases
AC	Activated carbon
AEL	Alkaline electrolysis
ATR	Autothermal reforming
BDD	Boron-doped diamond
DME	Dimethyl ether
DMF	Dimethylformamide
EV	Electrical vehicles
FT	Fischer-Tropsch
GDE	Gas diffusion electrodes
HER	Hydrogen evolution reaction
MECs	Microbial electrolysis cells
MeOH	Methanol
MFCs	Microbial fuel cells
MWCNTs	Multi wall carbon nanotubes
NCF	Nanoporous Cu film
NHE	Normal hydrogen electrode
NO_x_	Nitrogen oxydes
NPs	Nanoparticles
NTs	Nanotubes
PEM	Polymer electrolyte membrane electrolysis
PES	Polyethersulfone
POX	Partial oxidation
PPS	Polyphenylene sulfide
PTFE	Polytetrafluoroethylene
PtM	Power-to-Methane
PtL	Power-to-Liquids
PtX	Power-to-X
PV	Photovoltaic panels
RHE	Reversible hydrogen electrode
SCE	Saturated calomel electrode
SMR	Steam methane reforming
SNG	Substitute natural gas
SOEC	Solid oxide electrolysis
TOF	Turnover frequency
TON	Turnover number
WT	Wind turbines

Energy is considered one of the most important fundamental requirement for human survival and therefore, making this transition represents colossal modifications in our systems (The European Commission Directorate General for Energy, 2013; Overland, 2016). Energy is used to produce electricity, heat, cold, and a large amount of it is used as well for transportation, ranging from individual cars to planes, trains and boats (Thorin, 2014).

When specifically aiming at transportation fuels, the world demand is absolutely gigantic (2.5 billion tons of oil equivalent) (BP Energy Economics, 2018) although options are available and increasingly implemented around the world. In countries where green electricity is abundant (such as in some portions of Canada, namely Quebec), the opportunity for implementation of a larger pool of electric vehicles certainly has advantages (Ministère des Transports du Québec, 2015). Electric vehicles (EV) however, are still constrained by their capacities, limiting their widespread distribution in locations where population is largely distributed on a wide territory (Egbue and Long, 2012; Quak et al., 2016; Vassileva and Campillo, 2017). Other initiatives can include the production of biofuels from renewable carbon sources or biofuels. The latter are usually classified in three different “generations” where the first generally involves the utilization of ethanol (produced from grain or sugar-rich plants) as oxygenate in gasoline and fatty acid methyl ester (biodiesel) as a partial replacement for diesel (Alalwan et al., 2019). The second generation is usually related to the utilization of non-edible sugars (such as cellulose) as a sugar source to replace the contested first-generation feedstocks (Alalwan et al., 2019). Significant efforts have also been dedicated in converting all sorts of residual carbon sources such as waste plastics, MSW, residual agricultural and residual forest biomass to alkanes through techniques such as pyrolysis, gasification and liquefaction which all share their opportunities and challenges (Corma et al., 2011; Galadima and Muraza, 2015; Das and Tiwari, 2018; Kassargy et al., 2018). While it might be challenging for one of the previously mentioned option to cope for all the demand in transportation over the next decade, combining different opportunities may lead to good results and in term significantly reduce GHG emissions. Natural gas (NG) also represents a finite opportunity for the transportation sector since natural gas could represent a significant source of carbon (Fortis, 2018; Rego de Vasconcelos and Lavoie, 2018; Thiruvengadam et al., 2018) although the latter is still fossil based it is generally considered as a more sustainable option than classical oil (Rego de Vasconcelos and Lavoie, 2018; Thiruvengadam et al., 2018) and could share part of demand in a nearby future. NG can be used directly in some engine or can be transformed to gasoline, diesel and jet fuel through reforming and Fischer-Tropsch synthesis.

Transitioning from classical electricity production to sustainable models also represents its share of challenges (Lazkano et al., 2017). In opposition to traditional approaches such as nuclear, gas or coal power, renewable electricity production system such as photovoltaic panels (PV) and wind turbines (WT) is constrained by the alternating flux of electricity produced. While the production is optimal during some peak periods, the latter don't often fit with the demand, which makes of relying on these sole options a rather risky option, especially if such sustainable options are to be implemented in large cities. Other sources of renewable electricity such as hydroelectricity represents a never-ending flow of current and in some specific cases (such as in Quebec, Canada), the quantity of green electricity produced exceed the local demand which could eventually represent opportunities for other locations^2^. However, transportation of electricity over large distances is actually costly in light of the significant losses that are encountered over long distances^2^^,^^3^. In addition, some of the production facilities generating this renewable electricity are already in remote locations on the globe, which in turn involves significant losses as a starter. Hence, green electricity, either flowing or peaking, is constrained by storage, which has become over the years a significant source of concern for industry and governments, unavoidably transferring to academia (Lazkano et al., 2017).

Mechanical storage of electricity has been known for decades and options as simple as using peak energy to pump water in an elevated basin to release it during peak hours have been investigated and even implemented (Steinmann, 2017). Such options, although simple, requires a suitable landscape as well as being criticized for their efficiency (Steinmann, 2017). Batteries are also widely spread across the globe and are omnipresent in our lives going from cell phones to computer and of course, electric cars (Huang, 2018). Although originating from a historical technology, battery have been improving over the years both on efficiency and size, without forgetting their price, which is still accessible to the average consumers (Child et al., 2018). It goes without saying that batteries will have their place in the upcoming mixed-energy system where we are heading, however some of the best technologies actually on the market relies on lithium ions which in turn still represents a finite element (Child et al., 2018; Huang, 2018). Recuperation of ion has become a major concern in this field (Huang, 2018) although still far from solving another big challenge, which would be to produce a battery big enough to sustain a full large-scale city (Tervo et al., 2018).

Batteries allow the conversion of electrical energy toward chemical energy, playing on the redox functionalities of ions in solution. However, other opportunities do exist for chemical storage of renewable energy and are now referred as Power-to-X technologies (Sternberg and Bardow, 2015; Vázquez et al., 2018). As the name implies, the concept evolves around converting power (electricity) to chemicals (X), which could be very diversified hence the utilization of the “X.” The most well-known technology for the production of chemicals out of electricity is water electrolysis producing hydrogen and oxygen. Hydrogen produced through electrolysis has been acclaimed as one of the optimally sustainable fuel since its combustion leads only to the production of water, which involves minimal impact on GHG emissions (Carmo et al., 2013; Buttler and Spliethoff, 2018; Chi and Yu, 2018). Production of hydrogen through electrolysis is still limited in many cases by issues related to cost and storage (Carmo et al., 2013; Buttler and Spliethoff, 2018; Chi and Yu, 2018).

PtX technologies can also involve carbon-based structures which would make them more compatible to the existing infrastructure both for transportation and for large-scale energy production. One especially abundant carbon-based feedstock suitable for PtX technologies is carbon dioxide (CO_2_), which in turn is also the actual focus of the worldwide climate change efforts (Sternberg and Bardow, 2015; Vázquez et al., 2018). Using electrocatalytic systems or through the utilization of hydrogen as reducing agent, CO_2_ can now be used for the production of simple C_1_ molecules such as methanol (Frese, 1991; Bellotti et al., 2017) and methane (Manthiram et al., 2014; Stangeland et al., 2017). Reports from open literature shows as well that PtX technologies could eventually lead to the production of gasoline (Wei et al., 2017), diesel (Han et al., 2017), and even jet fuel (Schmidt et al., 2016). Hence, these technologies would at the same time allow storage of renewable energy, while reducing carbon dioxide emissions at the source AND producing liquid transportation fuels.

In this work, the different advances in Power-to-X technologies are investigated and discussed involving both situations where electricity is used directly to reduce carbon dioxide to chemicals up to technologies relying on the utilization of hydrogen for the reduction of carbon dioxide into a commodity that would be easier to implement into the existing worldwide infrastructure. Despite the fact that these technologies in some cases may not be entirely competitive with classical fossil fuels, they still represent an unavoidable pathway toward sustainability and only by promoting the development of these technologies can we ever hope to balance the impact of our society on the environment. The following sections will present the recent advances in the technologies for hydrogen production from renewable energy as well as the developments in the production of key chemicals, such as formic acid, formaldehyde, methanol, methane, and alkanes via Power-to-X technology.

## Hydrogen

Hydrogen (H_2_) is a key element for the production of value-added products such as methanol, formic acid, formaldehyde and liquid fuels out of CO_2_. Methane can also be used to the same purpose. However, using H_2_ would be preferable from an economical point of view since it allows the direct production of chemical and fuels, in opposition to CH_4_, which allows the production of syngas (Kondratenko et al., 2013). H_2_ is also an important and versatile energy vector with a low heating value (LHV) of 119.9 MJ/kg, which is more than two times higher than the LHV of methane (Baykara, 2018).

This molecule is not available in pure state in the environment and requires to be synthesized. The main pathway currently employed at industrial scale for hydrogen production is a well-established process called steam methane reforming (SMR) in which natural gas (or other fossil fuel) is reacted at high temperature (>700°C) with water vapor to produce hydrogen in the presence of a metal-based catalyst (Equation 1) (Ferreira-Aparicio et al., 2005). This process has a conversion efficiency range of 65–75% (Abdalla et al., 2018; Baykara, 2018).

(1) $$\begin{array}{r} \text{C}{\text{H}}_{4}+{\text{H}}_{2}\text{O}⇄\text{CO}+{3\text{H}}_{2}\;{\Delta \text{H}}_{298\text{K}}=206\text{ kJ}/\text{mol} \end{array}$$

Partial oxidation (POX) and autothermal reforming (ATR) are also processes used for hydrogen production from fossil fuels. In the POX process, hydrocarbons are reacted with steam and oxygen at varying concentrations to produce hydrogen (Equation 2). In opposition to SMR, such technology can be operated without a catalyst. However, very high temperatures are required (>1000°C) in order to get a hydrocarbon conversion. Also, a lower H_2_/CO around 2 is obtained. This process has been reported to have an efficiency around 50% (Baykara, 2018).

(2) $$\begin{array}{r} \text{C}{\text{H}}_{4}+\frac{1}{2}{\text{O}}_{2}⇄\text{CO}+{2\text{H}}_{2}\;{\Delta \text{H}}_{298\text{K}}=-38\text{ kJ}/\text{mol} \end{array}$$

The autothermal reforming (ATR) is a combination of SMR and POX. In this process, the energy released during the POX step is used to cope to the endothermic part of the SMR step. This process is also operated at high temperatures generally between 950 and 1,100°C (Lavoie, 2014; Rego de Vasconcelos and Lavoie, 2018) and has an efficiency around 60–75% (Abdalla et al., 2018). None of these three processes produce pure hydrogen since carbon monoxide (CO) is also produced at a H_2_/CO ratio varying between 2 and 3. Thus, a second step involving a water-gas shift reaction (Equation 3) is required in order to convert the carbon monoxide into hydrogen.

(3) $$\begin{array}{r} \text{CO}+{\text{H}}_{2}\text{O}⇄{\text{CO}}_{2}+{2\text{H}}_{2}\;{\Delta \text{H}}_{298\text{K}}=-41\text{ kJ}/\text{mol} \end{array}$$

Hydrogen at a 97% purity can also be produced from coal gasification, where the Koppers-Totzek process is the leading technology (Baykara, 2018). In this process, a entrained flow gasifier is used to convert the carbon into a gas mixture composed of methane, hydrogen and carbon monoxide at temperatures around 1,600–1,900°C, temperature of which usually enhances the reaction rates^4^.

The “hydrogen economy” concept, where H_2_ is used as an energy vector, is however not new and was first mentioned in the 70's after an oil crisis (Ball and Weeda, 2015). However, mainly due to the low price of fossil fuels along with the high costs and technical challenges related to the use of renewable resources, fossil fuels remained the primary resource for hydrogen production. During the last few years, the interest in the topic has risen again mainly due to climate change issues showing the need to develop other energy scenarios as well as to progress on materials and renewable power generation (Hansen, 2015). However, in light of the actual environmental concerns, the advantages of using H_2_ as energy vector is still highly dependent on how it is produced (Ball and Weeda, 2015). There are different approaches currently under investigation for the sustainable production of H_2_, water electrolysis being the most promising one, which will be discussed in the following section. Other approaches to sustainably produce H_2_ are biomass electroreforming and the use of microorganisms in a bio-electrochemical approach. Table 2 summarizes the key operational parameters as well as the main advantages and disadvantages of each technology.

**Table 2:** Comparison between different processes for hydrogen production.

**Main techniques**	**Cell voltage**	**Power consumption (kWh/m^**3**^ H_**2**_)**	**T (°C)**	**P_**max**_ (bar)**	**Efficiency (%)**	**TRL**	**Advantages/Disadvantages**	**References**
**ELECTROLYSIS**								
Alkaline	1.8–2.4	3.8–8.2	<100	690	59–79	Commercial	**Advantages:** Low cost; mature technology; possible application in large plant sizes **Disadvantages:** Low current density; low dynamics; corrosive electrolyte	Dincer and Zamfirescu, 2016; Götz et al., 2016; Sapountzi et al., 2017; Buttler and Spliethoff, 2018
PEM	1.8–2.2	4.4–7.1	<150	400	62–82	Commercial	**Advantages:** High power density; high pressure; rapid system response; no corrosive substances **Disadvantages:** high cost; fast degradation of membranes	Dincer and Zamfirescu, 2016; Götz et al., 2016; Sapountzi et al., 2017; Buttler and Spliethoff, 2018
SOEC	–	3.7	>500	30	Up to 100	Prototype	**Advantages:** High efficiency; co-electrolysis of CO_2_ and steam; possible integration of waste heat **Disadvantages:** low long term cell stability; not suited for fluctuating systems	Dincer and Zamfirescu, 2016; Götz et al., 2016; Sapountzi et al., 2017; Buttler and Spliethoff, 2018
Microbial electrolysis	0.2	–	<55	P_atm_	–	Laboratory	**Advantages:** Use of organic waste as substrate; low energy consumption **Disadvantages:** design of efficient and scalable prototypes	Hu et al., 2008; Azwar et al., 2014
Biomass electro-reforming	<1	<2.4	<100	P_atm_	–	Laboratory	**Advantages:** Flexible feedstock; low energy demand **Disadvantages:** Low calorific value of biomass	Baykara, 2018; Coutanceau et al., 2018

### Water Electrolysis

Water electrolysis is the best known electrochemical process for producing hydrogen using renewable electricity (Dincer and Zamfirescu, 2016) and it will play a crucial role on the development of the hydrogen economy and of the PtX technology since it produces high-purity hydrogen suited not only for applications, such as metallurgical, fine chemicals and aerospace industry but also for hydrogen filling stations. This technology allows onsite H_2_ production from renewable energy, contributing to the use of H_2_ as an energy storage medium (Chi and Yu, 2018) as well as to the use of renewable H_2_ on the production of chemicals and fuels via PtX technology.

**Figure 1:**
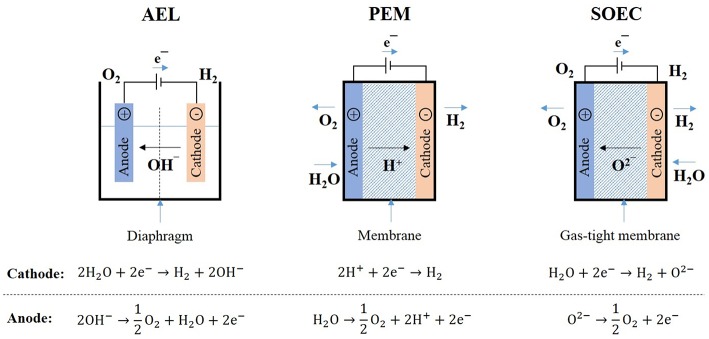
Technologies for water electrolysis.

There are three main electrolysis technologies used for hydrogen production classified according to the electrolyte (Figure 1): alkaline water electrolysis (AEL), polymer electrolyte membrane electrolysis (PEM) and solid oxide electrolysis (SOEC). AEL is already a mature technology with commercial large-scale systems. PEM systems are also commercially available but only for small scale hydrogen production while SOEC is still at prototype stage. These technologies are compared in Table 2. The following topics will briefly discuss the recent advances in each technology as well as their potential for PtX applications.

#### Alkaline Electrolysis (AEL)

Alkaline electrolysis is the most mature and reliable electrolysis technology and has been used at commercial MW-scale for decades with stacks currently available up to 6 MW (1,400 Nm^3^/h) (Carmo et al., 2013; Götz et al., 2016). This technology relies on the use of two electrodes immersed in a liquid alkaline electrolyte, usually KOH or NaOH solution, and separated by a diaphragm, which avoids the mixture of the product gases (Figure 1). Electrons flow from the anode to the cathode, where they are consumed by H^+^ ions to form H_2_. Hydroxide ions (OH^−^) are then transferred through the alkaline electrolyte solution from the cathode to the anode, where they are oxidized into oxygen and water (Zeng and Zhang, 2010). High purity H_2_ (up to 99.9%) can be obtained with this technology (Buttler and Spliethoff, 2018). O_2_ is also produced with purity in the range of 99–99.8%, which can be increased to 99.999% using catalytic purification (Buttler and Spliethoff, 2018). The electrolyte concentration usually varies between 25 and 30 wt.% for working temperatures in the range of 70–100°C and for typical pressures between 1 and 30 bars (Coutanceau et al., 2018). This type of electrolyser can either operate at atmospheric or high pressure, with high-pressure electrolysers operating up to 690 bar (Table 2) (Dincer and Zamfirescu, 2016). The production of pressurized hydrogen is particularly interesting due to its higher energy efficiency when compared to H_2_ pressurization after production, which is usually required to further use the hydrogen produced or for direct grid injection (Götz et al., 2016). According to Voitic et al. (2015), H_2_ needs to be compressed up to 500 bars for transportation purposes as well as filling stations. Moreover, the gas compression along with the transportation and storage of hydrogen represent a significant cost in the hydrogen production chain (Voitic et al., 2015). Nevertheless, pressurized electrolysers have lower efficiency and produce lower purity hydrogen when compared to atmospheric pressure electrolysers (Götz et al., 2016).

The main advantages of using AEL systems are the readily availability, the durability (55,000–120,000 h) as well as the use of mature stack components (Schmidt et al., 2017). This technology has also the advantage of low capital costs when compared to PEM and SOEC, having investments costs around 800–1,500 €/kW and maintenance costs of 2–3% of the annual investment costs (Buttler and Spliethoff, 2018). Some PtG plants rely on AEL for H_2_ production. The Audi e-gas plant, the world's biggest PtG plant, produces H_2_ with three AEL with a total power of 6 MW^5^ (Lambert, 2018). Similarly, the BioCatProject, which is a partnership between companies, such as Electrochaea, Audi and Hydrogenics, is currently developing a PtG plant using a 1 MW AEL electrolyser for H_2_ production^6^ (Götz et al., 2016).

Despite being a well-stablished technology, AEL has a few drawbacks, such as low partial load range and limited current density, which have a negative impact on the hydrogen production costs (Carmo et al., 2013; Schmidt et al., 2017). AEL electrolysers also have a limited dynamic operation, which renders them difficult to adapt to variable renewable energy sources, such as solar and wind power (Chi and Yu, 2018) and decrease the system efficiency as well as the gas purity (Schmidt et al., 2017).

Recent studies on AEL electrolysis have mainly focused on the development of new diaphragm and electrode materials to improve the performance of the system (Coutanceau et al., 2018). The development of new diaphragm materials have taken into consideration features, such as performance, cost and health hazard (Ohmori et al., 2007). Asbestos was initially used as diaphragm. However, it has been replaced by other materials, such as polytetrafluoroethylene (PTFE), reinforced polyethersulfone (PES) membranes, glass reinforced polyphenylene sulfide (PPS) compounds, nickel oxide layer on a mesh with titatinum oxide and potassium titanate, especially due to its health risks (Rashid et al., 2015; Coutanceau et al., 2018). The electrode shape, composition and electronic properties have also been studied aiming to enhance and stabilize the electrode activity. Modifications on the electrode surface, such as addition of slits and holes, have shown to help the dissemination of gas bubbles, which are one of the major causes for extra ohmic losses (Zeng and Zhang, 2010). Nickel is the most used electrode material due to its high activity and stability in alkaline media and it has been used in commercial systems (Zeng and Zhang, 2010; Coutanceau et al., 2018). Addition of iron has proved to increase the stability of nickel electrodes by preventing the formation of nickel hydride phase at the surface of the electrode. Noble metals, such as platinum (Pt) and ruthenium (Ru) are also used as electrocatalysts and are known to improve the activity for the oxygen evolution reaction (Zeng and Zhang, 2010). In summary, the electrode activity can be improved by using different transition and/or noble metals as well as alloys and physical modifications of the electrode can improve gas removal and decrease the ohmic losses. Further studies are still required to reduce the overpotential of the two half reactions, to improve the electron and ionic transfer as well as to reduce the ohmic losses.

#### Polymer Electrolyte Membrane Electrolysis (PEM)

In this type of electrolyser, the two half-cells are separated by a proton exchange membrane, often made of Nafion polymer (Figure 1). Water is split into oxygen (O_2_), protons (H^+^), and electrons. H^+^ are then transferred from the anode to the cathode through the proton exchange membrane and electrons flow from the anode to the cathode via external direct current (DC) power source. Protons and electrons then recombine at the cathode to produce hydrogen (H_2_) (Martinson et al., 2014). The polymer electrolyte membrane has the role of lowering the gas crossover, providing high proton conductivity and allowing high pressure operations as well as Faraday efficiency close to 100% (Carmo et al., 2013; Ogawa et al., 2018). Since the gas crossover is limited in this type of electrolyser, high purity hydrogen can be produced. Using proton exchange membranes instead of liquid electrolytes allows a quick response to the power input and hence the use of a wide range of power input (Carmo et al., 2013). The systems are usually operated at temperatures lower than 150°C and with typical pressures between 20 and 50 bar (Table 2). High pressure systems can work up to 400 bar (Dincer and Zamfirescu, 2016). This technology has become very promising for H_2_ production due to its compact design, high efficiency and high H_2_ output pressure (Chi and Yu, 2018). It also provides a better coupling with intermittent systems as compared to AEL and SOEC technologies (Götz et al., 2016), making it a good candidate for PtX applications. Due to the its potential, companies, such as Siemens, Proton OnSite, Hydrogenics, AREVA H_2_Gen etc., have invested in development of this technology (Bessarabov and Millet, 2018). A recent joint venture between Enbridge and Hydrogenics has taken this technology one step further in a 2.5 MW Power-to-Gas facility. The Markham Energy Storage facility built in Canada uses a 1.25 MW PEM electrolyser to produce H_2_ that can further be stored, shipped directly to refueling stations or to industrial and commercial customers^7^ (Core, 2018). Air Liquide has also recently announced the construction in Canada of a 20 MW PEM electrolyser for zero-carbon hydrogen production using Hydrogenics technology. This unit will be the world's largest PEM electrolyser^8^.

The major drawbacks of this technology are related to the high costs of materials and components that need to resist the low pH conditions as well as the high over voltages (Carmo et al., 2013). These conditions lead to the use of expensive titanium-based components as well as of noble metals-based electrocatalysts, such as platinum and iridium (Götz et al., 2016; Chi and Yu, 2018). According to Buttler and Spliethoff (2018), investment costs for these systems vary between 1,400 and 2,100 €/kW while the maintenance costs represent 3–5% of the annual investment costs.

Literature on PEM electrolysis is not abundant. However, the possibility of coupling this technology to intermittent renewable energy has led to an increase of the number of studies in the topic, which have focused on the reduction of the high costs related to this technology and more specifically on the design and synthesis of electrocatalysts. Few studies have also focused on the improvement of materials for current collectors and separator plates (Carmo et al., 2013). The replacement of noble metals-based electrocatalysts as well as decreasing the noble metal content are approaches current under investigation. Transition metals-based materials, such as molybdenum sulfide, cobalt sulfide, nickel-molybdenum alloys, iron, and cobalt phosphates etc., have been investigated as promising low cost alternatives to noble metals electrocatalysts (Di Giovanni et al., 2016). Addition of low cost oxides, such as SnO_2_, Nb_2_O_5_, and TiO_2_ to noble metals-based electrocatalysts, such as IrO_2_ and RuO_2_, has also been reported aiming to reduce the noble metal loading while maintaining similar catalytic and electronic conductivity properties (Datta et al., 2013). Finally, different synthesis methods have also being investigated to enhance the catalysts performance as well as to reduce the precious metal loading (Chourashiya and Urakawa, 2017). In summary, in order to this technology to achieve large scale, challenges related to the use of expensive catalysts and to low corrosion resistance and high cost collector and separator plates still need to be overcome (Carmo et al., 2013).

#### Solid Oxide Electrolysis (SOEC)

In this technology, the solid oxide electrolysis cell operates at high temperatures (700–1,000°C), reducing the equilibrium cell voltage and thus the electricity demand to lower than 4 kWh/Nm^3^ H_2_ (Table 2) (Götz et al., 2016). SOEC electrolysis is in fact the reverse process occurring in a fuel cell (Hansen, 2015), where H_2_O (steam) reacts at the cathode with electrons from an external power source, producing H_2_ and oxygen ions which are transported through a gas-tight membrane to the anode, where oxygen ions combine to form O_2_ and liberate electrons (Figure 1). Hydrogen is produced in this process at high Faraday efficiency around 100% (Ogawa et al., 2018). Another advantage of this technology is the possibility of heat integration with exothermic processes, such as methanol production. Heat released from these processes can be used to heat the steam to the temperature of the SOEC process (Buttler and Spliethoff, 2018). SOEC systems also offer the possibility of flexible operation between electrolysis and fuel cells. Since H_2_ produced through this process could be later reconverted in electricity using a fuel cell, this technology could represent an opportunity to store renewable electricity surplus generated by wind or solar power, for example. SOEC also presents the capacity of co-electrolysis of CO_2_ and steam for syngas production, which can be later converted into value-added products, such as liquid fuels. This capacity of co-electrolysis renders this technology very attractive for Power-to-X applications.

Contrarily to AEL and PEM, which are already at commercial level, SOEC is still at prototype stage and parameters, such as lifetime, cycling stability and pressurized operation still need to be validated (Buttler and Spliethoff, 2018). The major drawbacks preventing this technology from reaching large scale is the fast material degradation and thus low stability (Buttler and Spliethoff, 2018) related to the high temperature used and to long-term operation. Studies have proven that this degradation can be limited when the current density is low (<1 Acm^−2^). Hence, recent studies have focused on improving the stability of materials at high current densities (Ogawa et al., 2018).

Electrode materials must be ionic and electronic conducting in order to facilitate electron and mass transport as well as to allow the migration of O^2−^ species (Coutanceau et al., 2018; Arunkumar et al., 2019). In general, electrode materials consist of mixed oxides with perovskite structure. Ni-YSZ (yttria-stabilized zirconia) and LSM (lanthanum strontium manganite) are the most used materials for cathode and anode, respectively (Moçoteguy and Brisse, 2013; Ogawa et al., 2018). LSM has a coefficient of thermal expansion close to the one of the electrolyte, stabilizing the electrolysis cell. Moreover, LSM has very low chemical reactivity with YSZ, increasing the lifetime of the material (Moçoteguy and Brisse, 2013). Double perovskites, fluorites and metals have also been studied (Arunkumar et al., 2019). Mixing different oxides at different amounts has shown to improve activity and stability of the materials. Adding Co_3_O_4_ to LSM-BCZYZ, for example, significantly improve the electrode processes and enhances the current density under a certain voltage (Li et al., 2013). Mixed ion-electron conducting electrodes, such as lanthanum strontium copper ferrite and lanthanum strontium cobalt ferrite have also been used to improve the ionic conductivity property of electrodes (Moçoteguy and Brisse, 2013).

Electrolytes are generally composed by zirconia-based materials doped with CaO, MgO, Sc_2_O_3_ as well as rare-earth oxides. However, the most used electrolyte is yttria-stabilized zirconia (YSZ) due to its good ionic conductivity and mechanical properties (Hansen, 2015). La_1−x_Sr_x_Ga_1−y_Mg_y_O_2.85_ (LSGM) has been identified as a promising electrolyte material due to its high ion conductivity that can be five times higher than that of classical YSZ. However, further investigation is still needed to prove the durability of this material (Moçoteguy and Brisse, 2013).

The flexibility regarding the operation mode of this technology, the possibility of CO_2_ and steam co-electrolysis as well as the higher energy efficiency of SOEC when compared to AEL and PEM has led major companies to invest in the development of SOEC. Sunfire, for example, has recently investigated this possibility by developing a SOEC module in a demonstration project at Salzgitter Flachstahl GmbH aiming to evaluate the potential of the module for energy balancing and load management. The module has a hydrogen production capacity of 40 Nm^3^/h and with an input power of 150 kW. The electrical efficiency of the system is higher than 80%. The system can also be reversed into fuel cell with an output power of 30 kW (Berkeley, 2017). Haldor Topsoe^9^, Fuel Cell Energy^10^ and Toshiba^11^ have also worked on the development of this technology.

### Other Approaches

#### Biomass Electroreforming

Biomass electroreforming for hydrogen production has gained increased attention due to its renewable character and to the flexibility of the feedstock. Different feedstock, such as energy crops and forestry and agricultural residue, can be used. In this process, organic compounds originated from biomass, such as alcohols and sugars, are oxidized in aqueous media coproducing hydrogen in the cathode of the electrolysis cell. The advantages of this process are the use of much lower temperatures (<100°C) then the classical routes (steam reforming and partial oxidation) and the production of high-purity hydrogen (Coutanceau et al., 2018). This process is also less energy-intensive than traditional water electrolysis process (Gutierrez-Guerra et al., 2015). Gutierrez-Guerra et al. (2015) compared the performance of the electrochemical reforming of ethanol with the classical catalytic reforming of ethanol. They reported that the electrochemical reforming process provided pure hydrogen in a single step while the catalytic process required additional steps. Moreover, the energy consumption and the amount of feedstock material were lower for the electrochemical process. Caravaca et al. (2013) investigated the hydrogen production from the electrochemical reforming of a bio-ethanol/water solution at 80°C in a PEM electrolyser. They showed that the electrochemical reforming of bio-ethanol led to the production of hydrogen and that the PEM electrolyser could perform for long operation times (6 h).

#### Microorganisms

Bio-electrochemical systems use microorganisms to catalyze the oxidation-reduction reaction at the cathodes and anodes. These systems are divided into Microbial Fuel Cells (MFCs) and Microbial Electrolysis Cells (MECs). In both processes, the bacteria decompose the organic material at the anode and hydrogen is produced at the cathode. However, additional electricity is required to the MEC system in order to supress the production of methane and oxygen, which lower the selectivity to H_2_ (Baykara, 2018). Despite the promising aspects of this technology, the design of efficient and scalable prototypes are still pending (Azwar et al., 2014).

## CO_2_ Derived Chemicals

**Figure 2:**
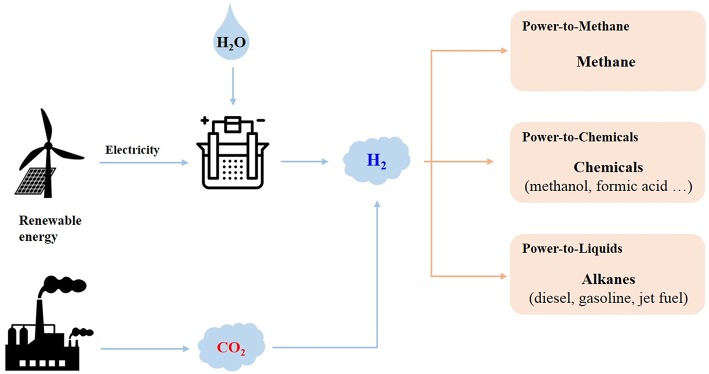
Power-to-X via CO_2_ hydrogenation.

**Figure 3:**
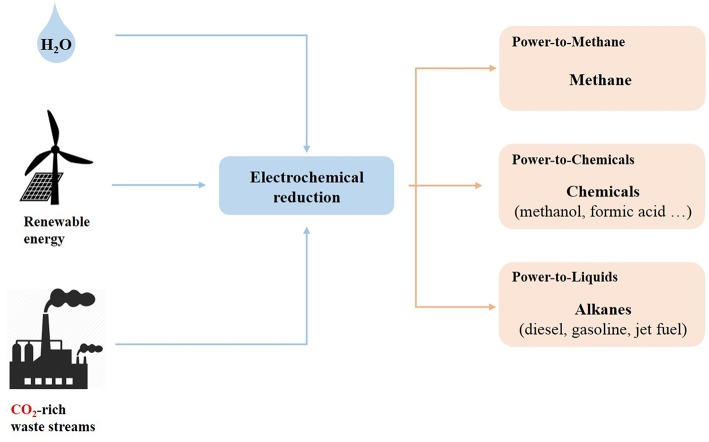
Power-to-X via electrochemical reduction.

PtX technologies can be used to produce value-added chemicals and fuels from CO_2_ through two main approaches: CO_2_ hydrogenation (Figure 2) and CO_2_ electrochemical reduction (Figure 3).

CO_2_ hydrogenation process (Figure 2) is performed in two steps, first of which, H_2_ is produced using renewable energies, such as solar, wind and hydro through process, such as water electrolysis. In the second step, the H_2_ produced is used to convert the CO_2_ issued from industrial processes or power plants, for example. This approach has gained increased attention due to its fast kinetics when compared to other approaches such as electrocatalysis and photocatalysis, and to its flexibility (Li et al., 2018b). However, this technology still faces some challenges, in particular the sustainable hydrogen production. As presented in the section Water Electrolysis, water electrolysis is the main technology investigated for sustainable hydrogen production. However, improvements in efficiency and cost reduction are still required to overcome technical and economic barriers to its commercialization (Götz et al., 2016). Finally, integration of the different steps of the technology (CO_2_ capture, water electrolysis and CO_2_ hydrogenation) is also still an issue.

CO_2_ electrochemical reduction (Figure 3) is a more recent and less advanced approach when compared to CO_2_ hydrogenation. In this process, CO_2_ reduction is connected to an oxidation reaction (usually the water oxidation), where water is oxidized in O_2_, protons (H^+^) and electrons (e^−^) at the anode. Then, the electrons flow to the cathode where they combine with CO_2_ to form different reduced products, such as methanol, methane and formic acid. In this case, renewable electricity is used to convert CO_2_ directly into fuels and chemicals, which represents a great potential for renewable energy storage and for lowering the GHG emissions into the atmosphere. In addition to its environmental advantages, the electrochemical reduction of CO_2_ can be performed at ambient temperature and pressure and can lead to the desired products only by adjusting some parameters such as the electrocatalyst, the operating potential and the electrolyte (Zhang and Zhang, 2017). Furthermore, the systems used with this processes are compact and could easily be scaled-up (Chen and Liu, 2018), leading the technology close to the demonstration phase (Reis Machado and Nunes da Ponte, 2018). However, considerable challenges such as high overpotentials, the slow kinetics of CO_2_ electroreduction, and poor product selectivity could slow down the use this technology at large scale (Kortlever et al., 2015; Chen and Liu, 2018). In addition, the direct electrochemical reduction of CO_2_ generates many different products, depending mainly on the reaction medium and on the catalyst (Albo et al., 2015). Hence, the development of electrocatalysts, allowing an efficient and selective reduction of the CO_2_ is essential for the development of this technology at large scale.

Among the many useful chemicals that can be synthesized directly from CO_2_, methane, methanol, alkanes, formic acid and formaldehyde, have a strategic use in the chemical industry, either as platform chemicals and/or for the production/storage of energy. The following topics intend to investigate the recent advances in the synthesis of the chemicals using both CO_2_ hydrogenation and CO_2_ electrocatalytically reduction.

### Formic Acid

Formic acid (HCOOH) is one of the main products that can be produced from CO_2_ reduction having a market value around 500–900 US$/mt HCOOH (Huang et al., 2018) as well, it is considered as a C_1_ building block and can also be used as a fuel in formic acid fuels cells (DFAFCs) (Mura et al., 2012; Kortlever et al., 2015; Huang et al., 2018). Its production is expected to reach 760 Mt by 2019 (Huang et al., 2018). Formic acid is also considered as one of the most promising and safest liquid hydrogen carriers (Onishi et al., 2018) with a hydrogen content of 52 g/L, whose release from formic acid is favored at room temperature (Δ*G*° = −32.8 kJ/mol) (Singh et al., 2016). The most common industrial process for formic acid production involves a two-step process, in which methyl formate is synthesized from methanol and carbon monoxide in the first step (Equation 4) and then, methyl formate is hydrolyzed into formic acid in the second step (Equation 5) (Singh et al., 2016; Huang et al., 2018). However, production of formic acid in the second step is not thermodynamically favorable.

(4) $$\begin{array}{r} \text{C}{\text{H}}_{3}\text{OH}+\text{CO}⇄\text{C}{\text{H}}_{3}\text{COOH }{\Delta \text{H}}_{\text{r}}=-29\text{ kJ}/\text{mol} \end{array}$$

(5) $$\begin{array}{r} \text{C}{\text{H}}_{3}\text{COOH}+{\text{H}}_{2}\text{O}⇄\text{HC}{\text{O}}_{2}\text{H}+\text{C}{\text{H}}_{3}\text{OH }{\Delta \text{H}}_{\text{r}}=+16.3\text{ kJ}/\text{mol} \end{array}$$

Formic acid can also be chemically synthesized by several other processes, such as oxidation of biomass, CO_2_ hydrogenation, CO_2_ electrochemical reduction, CO_2_ reduction by biocatalysts and also as a by-product of acetic acid production (Singh et al., 2016). This review will focus only on CO_2_ hydrogenation and on the CO_2_ electrochemical reduction.

#### CO_2_ Electrochemical Reduction

As showed in Equations (4) and (5), the equilibrium toward formic acid production is unfavorable in the classical process, leading to the investigation of other possible processes to the direct synthesis of formic acid. The electrochemical reduction of CO_2_ gained increased attention in the last decades due to its environmental advantages as well as its potential to directly convert CO_2_ into formic acid. However, as for all CO_2_ electrochemical reduction processes, high overpotentials and low product selectivity are the main bottlenecks for the development of this process (Reis Machado and Nunes da Ponte, 2018).

Thus, the development of less energy-intensive electrocatalysts is of importance to lower the overpotentials as well as to increase the selectivity to formic acid (Benson et al., 2009). Hori (2008) investigated the capability of different metals, such as Pb, Hg, Tl, In, Sn, Au, Ag, Zn, Pd, Ga, Cu, Ni, Fe, Pt, Ti to electrochemically reduce CO_2_ to formic acid. They found that the product distribution was deeply dependant on the metal electrodes. Pb, Hg, Tl, In, Sn, Cd, and Bi showed to be very efficient catalysts for the electrochemical reduction of CO_2_ in formic acid with Faradaic efficiencies up to 99.5%. However, they required very negative overpotentials around −2.0 V vs. saturated calomel electrode (SCE) to reach good product selectivity (Hori, 2008). Pt and Pd showed improved selectivity to formic acid, however, they also catalyzed the hydrogen evolution reaction (HER), a side reaction to CO_2_ reduction that lowers the Faradaic efficiency toward the formic acid production. CO poisoning is also a problem for Pt electrodes since the CO formed during the CO_2_ reduction covers the platinum surface, reducing the performance of the catalyst. However, Kortlever et al. (2015) reported that a palladium-platinum catalytic system reduced CO_2_ to formic acid using very low overpotentials, starting from −0.05 V vs. reversible hydrogen electrode (RHE) at pH = 6.7. Nevertheless, the main challenge remains to avoid the CO poisoning.

Since sulfur poisoning has been reported to be a considerable issue for HER catalysts it could be beneficial for competitive reactions. Thus, Huang et al. (2018) studied the influence of sulfur dopants in the performance of copper catalysts, aiming to limit the HER during CO_2_ electrochemical reduction to formic acid. They showed that a copper-based catalyst, doped with 2.7 wt.% of sulfur, exhibited a formate current density 46 times higher than the undoped catalyst. The sulfur-doped catalyst showed a faradaic efficiency of 75% during 12 h, while its structure was shown to play a role in the selectivity to formic acid. Kumar et al. (2017) evaluated the performance of Sn-based catalysts on the CO_2_ electrochemical reduction and they proved that a nanoporous and high-density grain boundary structure is an important factor to increase the selectivity and the rate of formic acid production from CO_2_ electroreduction. The SnO_2_ porous nanowires synthesized showed a CO_2_-to-HCOOH efficiency of 56%, being one of the most performing catalysts reported for this process.

Biocatalysts have also been used for the production of formic acid from CO_2_ electrochemical reduction, presenting higher selectivity than conventional metal electrodes. Hwang et al. (2015) investigated the use of different Methylobacteria as a whole-cell biocatalysts for the electrochemical conversion of CO_2_ to formate. They found that the *Methylobacterium extorquens* AM1 had a high capability to convert CO_2_ by suppling electrons, producing 60 mM of formate without requiring any additional hydrogen supply. However, the synthesis was relatively slow, taking 80 h to produce the 60 mM of formate with 1.9 g of the catalyst. Similarly, Le et al. (2018) investigated the performance of the *Shewanella oneidensis* MR-1 due to it powerful electron transfer system. With an optimization of the *S. oneidensis* MR-1 growth, up to 136.84 mM of formate was produced after 72 h. The reaction rate was 3.8 mM h^−1^ g^−1^, being almost 10 times faster when compared to the values previously obtained by Hwang et al. (2015).

#### CO_2_ Hydrogenation

The actual industrial process for formic acid production from methanol and carbon monoxide (Equations 4 and 5) emits around 3,100 kg of CO_2_ for each ton of formic acid produced. CO_2_ hydrogenation to formic acid (Equation 6) has gained increased attention since it could reduce the greenhouse gases emissions related to the formic acid production (Gunasekar Hariyanandam et al., 2016) by a 10-fold, especially if coupled with a hydrogen production process using renewable energy, such as electrolysis. Moreover, this process has become a significant milestone to consolidate formic acid as a reversible hydrogen storage carrier (Singh et al., 2016). The hydrogenation of CO_2_ in gas phase is not entropically favored though, since it involves the conversion of two gaseous reactants into liquid products (Wang et al., 2015; Gunasekar Hariyanandam et al., 2016). However, the reaction is favorable in aqueous medium (Equation 7).

(6) $$\begin{array}{r} \text{C}{\text{O}}_{2\left(\text{g}\right)}+{\text{H}}_{2\left(\text{g}\right)}⇌\text{ HCOO}{\text{H}}_{\left(l\right)}\;{\Delta \text{G}}_{298\text{K}}=32.8\text{ kJ}/\text{mol} \end{array}$$

(7) $$\begin{array}{r} \text{C}{\text{O}}_{2\left(\text{aq}\right)}+{\text{H}}_{2\left(\text{aq}\right)}⇌\text{ HCOO}{\text{H}}_{\left(aq\right)}\;{\Delta \text{G}}_{298\text{K}}=-4\text{ kJ}/\text{mol} \end{array}$$

Hence, the selection of the medium (solvents, water) is of crucial importance since it can allow for the reaction to be more thermodynamically favorable (Wang et al., 2015; Gunasekar Hariyanandam et al., 2016; Singh et al., 2016). In addition, basic additives, such as trimethylamine and ammonia are often used to shift the equilibrium toward formic acid production (Wang et al., 2015; Gunasekar Hariyanandam et al., 2016; Álvarez et al., 2017). Noble metals homogeneous catalysts are more commonly used in this process due to their promising catalytic performances. Noble metals, such as Ruthenium (Ru), Rhodium (Rd), and Iridium (Ir) have shown excellent catalytic results and transition metals, such as Nickel (Ni), Copper (Cu), and Iron (Fe) have also been investigated despite their lower activity due to their lower cost compared to the nobles metals (Wang et al., 2015; Gunasekar Hariyanandam et al., 2016; Álvarez et al., 2017).

Tanaka et al. (2009) studied the performance of a Ir(III)-PNP pincer complex in aqueous KOH medium for the hydrogenation of CO_2_ to formic acid. At 120°C and 6 MPa, the catalyst was very performant, leading to a high TOF of 73,000 h^−1^ for 48 h. They also showed that the use of a stronger base (KOH instead of K_3_PO_4_) could increase formic acid yield from 60 to 70%, confirming the strong role of the base in the reaction. Liu et al. (2015) developed an efficient Ir complex containing imine-diphosphine ligands for CO_2_ hydrogenation. The catalyst exhibited a TOF of 450,000 h^−1^ at 140°C for 20 h in a 5 M KOH medium. The catalytic performance of the Ir complex was attributed to the *C* = *N* bond of the ligand that acted as an acceptor of H_2_ that further led to the production of formate. Munshi et al. (2002) studied the influence of different bases and alcohols on the rate of supercritical CO_2_ hydrogenation using RuCl(OAc)(PMe_3_)_4_, a ruthenium trimethylphosphine complex. The results revealed that the selection of the appropriate amine and alcohol have a great influence on the rate of the reaction. The use of pentafluorophenol as alcohol and triethylamine as base at 50°C and 19 MPa for 20 min led to a TOF for formic acid production of 95,000 h^−1^. The pentafluorophenol alcohol, for example, could have acted either as a hydrogen donor or as a proton donor, favoring the hydrogenation reaction. To the best of our knowledge, Filonenko et al. (2014) developed a ruthenium complex catalyst that has showed the best results for formic acid production. They investigated the performance of a Ru-PNP-pincer catalyst in a batch reactor at 120°C and 2.7 MPa for 1 h using dimethylformamide (DMF) as solvent and 1,8-diazabicyclo[5.4.0]undec-7-ene (DBU) as base and obtained a TOF for formic acid production of 1,100,000 h^−1^. In agreement with the finding of Tanaka et al. (2009), they stated that strong bases play an important role in this reaction by affecting the rate determining step of the reaction. When a strong base is used, the initial H_2_ recombination is the rate-determining step.

Despite their excellent catalytic performances for hydrogenation of CO_2_ into formic acid, these homogeneous catalysts are difficult to separate from the products at the end of the reaction and the amount of CO_2_ actually hydrogenated per unit of time is still low, hindering their use at large scale (Gunasekar Hariyanandam et al., 2016; Álvarez et al., 2017). Moreover, these homogeneous catalysts may also promote the reverse reaction, in which the formate produced can be transformed back into CO_2_ and H_2_ (Gunasekar Hariyanandam et al., 2016). To cope with the problematics related to homogeneous catalysts, studies were recently published on the use of heterogeneous catalysts for the hydrogenation of CO_2_.

Umegaki et al. (2016) were the first to study the performance of ruthenium (Ru) nanoparticles (unsupported catalyst) in the hydrogenation of supercritical CO_2_ into formic acid. The reaction was carried out at 353 K and 13 MPa for 3 h in a mixture of water and trimethylamine. They observed that the metallic nanoparticles were very active and stable during the test, leading to a TOF of formic acid of 2,117 h^−1^. The stability of the catalyst was attributed to the fact that the nanoparticles maintained their metallic state after the test.

The influence of ruthenium-based catalysts doped on different supports, such as MgO, Al_2_O_3_, and activated carbon (AC) was studied by Hao et al. (2011). Each support was doped with 2 wt.% Rh and the hydrogenation tests were performed at 353 K and 13.55 MPa in the presence of ethanol as solvent and triethylamine as base, which were added to extract the formic acid, increasing the reaction rate. The Ru/AC and the Ru/Al_2_O_3_ catalysts showed good catalytic performances with turnover numbers (TON) of 10 and 91, respectively. The catalytic performance of the catalysts was attributed to the presence of hydroxyl groups on the surface of the catalysts that increased the adsorption of CO_2_. The higher yield of formic acid obtained with the Ru/Al_2_O_3_ catalyst compared to the Ru/AC was in fact due to its greater number of hydroxyl groups. The MgO support was chosen for its strong basic sites, eliminating the need of using basic additives that need to be neutralized and separated from the product at the end of the reaction. However, no formic acid was produced in the presence of this catalyst, probably due to the absence of hydroxyl groups on its surface as well as the high pH of the solution that lead to the formation of inactive RuO_2_ species. Similarly, Zhang et al. (2018) obtained a good catalytic performance of a Ru-based catalyst doped on an alumina support. They investigated the performance of the Ru-PPh_3_/Al_2_O_3_ catalyst as well as the presence of additives and solvents in the CO_2_ hydrogenation to formic acid reaction in a batch reactor at 80°C and 12 MPa for 1 h. The catalyst showed a high turnover frequency of formic acid of about 751 h^−1^ in a mixed solution of ethanol, triethylamine and water and in the presence of KH_2_PO_4_ and PPh_3_ as additives. They reported that the high TOF obtained was related to the use of PPh_3_ as proton donor, KH_2_PO_4_ as proton source and trimethylamine as basic additive, increasing the conversion of CO_2_.

Su et al. (2015) studied the performance of different heterogeneous palladium-based catalysts doped on different supports. In this work, all catalysts were doped with 5 wt.% of palladium (Pd) and the best hydrogenation results were obtained at 20°C for 1 h in a 1 M NH_4_HCO_3_ medium. They showed that the CO_2_ hydrogenation test performed at 2.7 MPa with the Pd/Al_2_O_3_ catalyst resulted in a TOF of 278 h^−1^ while the hydrogenation test, performed at 5.5MPa with the Pd/AC (activated carbon) nanocatalyst, showed a TOF as high as 1,103 h^−1^. The main reasons for the difference in the performance of both catalysts was the localized higher H_2_ concentrations on the surface of the activated carbon-based catalysts as well as a higher Pd dispersion that favored the hydrogenation reaction.

### Formaldehyde

Formaldehyde (CH_2_O) is currently used in about 50 industrial process as building block for the production of daily life products, such as paints, cosmetics, resins, polymers, plastics etc (Heim et al., 2016; Zhang et al., 2017). It is mainly produced via the energy and cost-intensive Formox process in which methanol is partially oxidized at 300–400°C (Equation 8) (Heim et al., 2016). Methanol used in the process is produced from syngas, which in turn is produced from steam reforming of natural gas (Heim et al., 2016). More than 35% of the world methanol production is actually used for formaldehyde production (Heim et al., 2017). The drawbacks of this process are high temperatures and energy-intensive compression and purification steps, affecting the overall economics of the process.

(8) $$\begin{array}{r} \text{C}{\text{H}}_{3}\text{OH}+{\frac{1}{2}\text{O}}_{2}⇄\text{HCHO}+{\text{H}}_{2}\text{O }{\Delta \text{H}}_{298\text{K}}=-159\text{ kJ}/\text{mol} \end{array}$$

Since CH_2_O is a hydrogen-rich molecule, it has a great potential to be used as a liquid hydrogen carrier, capable of delivering high-purity H_2_ in a hydrogen fuel cell (Heim et al., 2017). Moreover, it could be even more interesting as liquid hydrogen carrier than methanol since formaldehyde reforming process is much less energy intensive than methanol reforming (Heim et al., 2017). However, in order to produce H_2_ with a low carbon footprint, formaldehyde has to be produced more sustainably. There has been recent efforts for developing new processes for sustainably producing formaldehyde, such as CO_2_ hydrogenation, electrochemical and enzymatic approaches. However, the literature on this subject is relatively scarce, especially when heterogeneous catalysts are considered. The main findings on the formaldehyde production via CO_2_ reduction will be detailed in the following sub-sections.

#### CO_2_ Electrochemical Reduction

Very few reports from the open literature related the electrochemical reduction of CO_2_ to formaldehyde. Nakata et al. (2014) reported the highest Faradaic efficiency to formaldehyde obtained through CO_2_ electroreduction up to date. The authors investigated the CO_2_ reduction using Boron-doped diamond (BDD) electrodes in various electrolytes, such as methanol and seawater. When seawater was used as electrolyte, relatively low formaldehyde yield of 36% was obtained due to impurities in the seawater and to the narrow potential window in water. On the other hand, a Faradaic efficiency of 75% to formaldehyde was obtained at −1.5 V (vs. Ag/AgCl). When a glassy carbon electrode was used, a Faradaic efficiency of only 15% was obtained in the same conditions. The performance of the BDD electrodes was attributed to the presence of sp^3^-bonded carbon, while glassy carbon has large amounts of sp^2^-bonded carbon, resulting in low Faradaic efficiencies. Furthermore, BDD electrodes presented very good stability over 20 h, while the surface of the glass carbon electrode dramatically changed after 20 h of electrolysis, highlighting the potential of the BDD electrodes to formaldehyde production via CO_2_ electrochemical reduction.

#### CO_2_ Hydrogenation

Similarly to the electrochemical reduction, there are only few reports in the literature on CO_2_ hydrogenation to formaldehyde using heterogeneous catalysts (Heim et al., 2017). Lee et al. (2001) studied the production of formaldehyde via CO_2_ hydrogenation over a PtCu/SiO_2_ catalyst at 423 K and 600 kPa and varying the H_2_/CO_2_ feed ratio between 3 and 20. The authors reported that the rate of formaldehyde production increased considerably with increase of the H_2_/CO ratio, ranging from 0.21 × 10^−4^ mol min^−1^ ${\text{g}}_{\text{cat}}^{-1}$ at H_2_/CO = 3–0.87 × 10^−4^ mol min^−1^ ${\text{g}}_{\text{cat}}^{-1}$ at H_2_/CO = 20. Conversely, the rate of methanol production greatly decreased with an increase of the H_2_/CO ratio. These results led the authors to conclude that the relative concentration of surface hydrogen on the catalyst play a major role in the selective production of formaldehyde. For comparative purposes, Cu/SiO_2_ catalyst was also tested in the same conditions. However, in this case, when a H_2_/CO = 20 was used, the formaldehyde production was negligible. The authors concluded that Pt played an important role in the adsorption of hydrogen, which would then migrate to the copper surface, promoting the CO_2_ hydrogenation into formaldehyde.

Chan et al. (2018) recently reported the production of formaldehyde from CO_2_ hydrogenation in aqueous solution, using formic acid as intermediate of the CO_2_ hydrogenation process. The performance of the Pt-Cu/γ-Al_2_O_3_, Pt-Ni/γ-Al_2_O_3_, Ru-Cu/γ-Al_2_O_3_, and Ru-Ni/γ-Al_2_O_3_ catalysts was evaluated at 70 bar and at a temperature range of 298–363 K. The authors reported that the conversion of formic acid into formaldehyde is possible, despite not been thermodynamically favored, with Pt-Cu/γ-Al_2_O_3_ being the catalyst with the highest formic acid conversion and formaldehyde yield (≈6 mmol L^−1^ ${\text{g}}_{\text{cat}}^{-1}$). However, all the formic acid converted was not transformed into formaldehyde. The authors suggested that this could be related to the thermal decomposition of formic acid. However, they did not evaluate the formation of other products to confirm this hypothesis.

### Methanol

Methanol (CH_3_OH) is one of the main commodities produced worldwide, with a price around 470 USD per metric ton (2018, Methanex)^12^. It is also an important feedstock in the chemical industry for the production of olefins, dimethyl ether and liquid fuels, thus becoming an interesting alternative to fossil fuels (Goeppert et al., 2014; Dang et al., 2018). Moreover, CH_3_OH is a liquid energy carrier, being easier to handle and transport than gases and solid materials (Onishi et al., 2018).

Methanol is currently produced at commercial scale from fossil fuel-based syngas in a two-step process. In the first step, syngas with a H_2_:CO ratio close to 3:1 is produced from steam reforming of natural gas (Equation 1). Then, after adjusting the H_2_:CO ratio to around 2:1, the syngas is converted into methanol using copper-based catalysts (Equation 9) (Olah et al., 2009; Albo et al., 2015).

(9) $$\begin{array}{r} \text{CO}+{2\text{H}}_{2}⇄\text{C}{\text{H}}_{3}\text{OH }{\Delta \text{H}}_{298\text{K}}=-90.7\text{ kJ}/\text{mol} \end{array}$$

One of the simplest ways to sustainably obtain liquid products from CO_2_ is the production of methanol mainly electrochemically and through hydrogenation (Goeppert et al., 2014), two pathways that will be detailed in the following sections.

#### CO_2_ Electrochemical Reduction

As for most of the CO_2_ electrochemical reactions reported in this work, the development of catalysts able to selectively produce methanol still represents a challenge to be overcome. Copper and copper-based electrodes have proved to be one of the most performing materials for the electrochemical conversion of CO_2_ into alcohols, including methanol (Albo et al., 2015). Frese (1991) was one of the first to investigate the CO_2_ reduction to methanol over copper surfaces. The authors showed that an anodized Cu foil could successfully reduce CO_2_ into methanol using a 0.5 M KHCO_3_ as solution and −1.9 V (vs. SCE) at a rate as high as 10^−4^ mol cm^−2^ h^−1^. Faradaic efficiency for CH_3_OH reached about 240%. Efficiencies higher than 100% were obtained since the theoretical analysis considered six-electron reduction of CO_2_ to CH_3_OH and H_2_O and the HER also occurred. Similarly, Le et al. (2011) obtained a methanol production rate of 43 μmol cm^−2^ h^−1^ and Faradaic efficiency of 38% in 0.5 M KHCO_3_ using cuprous oxide thin films at −1.1 V (vs. SCE). The authors showed that this rate of methanol production as well as the Faradaic efficiency were much higher than those obtained with air-oxidized (0.9 μmol cm^−2^ h^−1^) and anodized (1.5 μmol cm^−2^ h^−1^) copper electrodes, suggesting that Cu (I) species might play a key role in the electrode activity and selectivity to methanol. Malik et al. (2016) studied the efficiency of multi wall carbon nanotubes (MWCNTs) impregnated with CuO_2_ to reduce CO_2_ into methanol, due to their excellent structural and electrical properties, making them good candidates for electrochemical applications. The authors reported that the MWCNTs acted as active sites for CO_2_ conversion as well as traps for electrons, increasing the rate of conversion of intermediates into methanol. Also, CuO_2_ loadings varying between 10 and 50 wt.% were investigated. 30% CuO_2_-MWCNTs catalyst showed the best catalytic performance, achieving 38% of Faradaic efficiency to methanol at −0.8 V (vs. Ag/AgCl) in a 0.5 M NaHCO_3_ medium. CuO_2_ loadings (higher than 40 wt.%) led to an agglomeration of the CuO_2_ particles and overall larger crystallite sizes, decreasing the surface area of the catalyst and the number of active sites.

The use of copper alloys have also been investigated, since it may enhance the electrochemical CO_2_ reduction to CH_3_OH. Actually, most of the industrial catalysts currently used for methanol production are composed of Cu-Zn mixed oxides, highlighting the synergetic effect of the metals to improve methanol production (Frese, 1991; Albo et al., 2015). Watanabe et al. (1991) evaluated the capacity of Cu-Ni, Cu-Sn, Cu-Pb, Cu-Zn, Cu-Cd, and Cu-Ag alloys to electrocatalytic reduce CO_2_ into methanol in a 0.05 M KHCO_3_ aqueous solution. They observed that the product distribution was dependant on the copper alloy. While a Cu-Ni alloy produced CH_3_OH and HCOOH, Cu-Sn, and Cu-Pb enhanced the production of HCOOH and CO. A Faradaic efficiency to methanol of 10% was obtained with the Cu-Ni alloy at −0.4 V (vs. Ag/AgCl) while no CH_3_OH was formed when pure Cu or Ni were used alone even when a wide potential range from −0.5 to −1.5 V was considered. They concluded that this difference in the catalytic performance was related to a mechanism involving the introduction of hydrogen atoms on the alloy surface by Ni sites. Jia et al. (2014) also investigated the performance of copper-based alloys. The authors prepared Cu-Au alloys through electrochemical deposition with a nanoporous Cu film (NCF) as template. The Cu_63.9_Au_36.1_/NCF alloy showed a Faradaic efficiency of methanol of 15.9%, which was around 19 times higher than that obtained with pure Cu. In fact, the Cu-Au alloy favored not only the CO_2_ reduction reaction but also the CO reduction, improving the overall conversion of CO_2_ into methanol.

Kuhl et al. (2014) further investigated the CO_2_ electrocatalytic reduction over different metals, such as Au, Ag, Zn, Cu, Ni, Pt, and Fe. Experiments were carried out in 0.1 M KHCO_3_ electrolyte and at different voltages varying between −1.6 and −0.4 V (vs. RHE). The best results were obtained with Au, presenting almost 100% of Faradaic efficiency to methanol. On the contrary, Fe showed to be inefficient for methanol production under these conditions, presenting 100% of current efficiency to methane production, instead of methanol. The other metals were able to produce both methanol and methane. The authors reported that one of the main parameters that influences the selectivity to methanol or methane is the oxophilicity (capacity to adsorb oxygen) of the catalyst surface. Au, metal with the lowest oxophilicity among the metals investigated, produced only methanol whereas Fe, metal with the highest oxophilicity, produced only methane.

Since the hydrogen evolution reaction (HER) competes with the CO_2_ reduction (hence lowering the Faradaic efficiency of the CO_2_ reduction reaction), Olah and Prakash (2010) investigated the possibility of using the HER as an advantage. The authors used a 0.1 M KHCO_3_ aqueous solution as electrolyte and water was electrolyzed while CO_2_ was reduced at an Au cathode at −3.2 V (vs. Ag/AgCl), producing syngas with a H_2_:CO ratio close to 2:1. They showed that the total Faradaic efficiency for H_2_ and CO was close to 100%. The syngas produced could then be converted into methanol using the same process that is currently used at industrial scale (Equation 9). The advantages of this process are the high Faradaic efficiencies for syngas production, absence of a purification step since no impurity is present and production of valuable high-purity oxygen at the anode (Olah et al., 2009).

Ru and RuO_2_ have also shown to be promising materials to for the electrocatalytic reduction of CO_2_ due to their high electrical conductivity, high electrochemical stability, intermediate hydrogen overpotential and capacity to reversibly adsorb hydrogen for CO_2_ reduction (Qu et al., 2005). Bandi (1990) reported that electrodes composed of 35% Ru and 65% TiO_2_ presented current efficiencies for methanol production as high as 24% when polarized near the equilibrium potential of hydrogen evolution in solutions of 0.2 M Na_2_SO_4_ saturated with CO_2_. The results led the authors to conclude that the first electron transfer is the rate-determining step in electrochemical reduction of CO_2_ on oxide surfaces at low pH. Finally, the authors suggested that the methanol efficiency could be improved by changing the oxide composition and preparation parameters. Qu et al. (2005) investigated the electrochemical CO_2_ reduction properties of RuO_2_/TiO_2_ nanoparticles (NPs) and nanotubes (NTs). RuO_2_/TiO_2_ NTs showed a Faradaic efficiency for CH_3_OH of 60.5% at −0.8 V (vs. SCE) in 0.5 M NaHCO_3_, which was about 20% higher than that obtained with RuO_2_/TiO_2_ NPs. The authors reached similar conclusions to Malik et al. (2016), suggesting that the surface structure of the nanotubes composite had an important role for achieving high efficiency and selectivity to the desired products. Table 3 summarizes the performance of different catalysts for CO_2_ electrochemical reduction into CH_3_OH reported in the literature.

**Table 3:** Summary of different electrocatalysts used for electrocatalytic reduction of CO_2_ in methanol.

**Catalyst**	**Medium (electrolyte)**	**E (V)**	**CH_**3**_OH production rate**	**Faradaic efficiency (%)**	**References**
Cu foil	0.5 M KHCO_3_	−1.9 V vs. SCE	10^−4^ mol cm^−2^ h^−1^	240	Frese, 1991
Cuprous oxide thin films	0.5 M NaHCO_3_	−1.1 vs. SCE	0.43 × 10^−4^ mol cm^−2^ h^−1^	38	Le et al., 2011
CuO_2_-MWCNTs	0.5 M NaHCO_3_	−0.8 vs. Ag/AgCl	–	38	Malik et al., 2016
Cu-Ni	0.05 M KHCO_3_	−0.4 V vs. Ag/AgCl	–	10	Watanabe et al., 1991
Cu_63.9_Au_36.1_/NCF	0.5 M KHCO_3_	−1.1 vs. SCE	–	15.9	Jia et al., 2014
Au	0.1 M KHCO_3_	−0.7 vs. REH	–	≈100	Kuhl et al., 2014
Pt RuO_2_/TiO_2_ NTs (nanotubes)	0.5 M NaHCO_3_	−0.8 vs. SCE	–	60.5	Qu et al., 2005
RuO_2_+TiO_2_	0.05 M H_2_SO_4_	−0.9 vs. Hg_2_SO_4_	–	24	Bandi, 1990

#### CO_2_ Hydrogenation

The most direct way to produce methanol from CO_2_ via a PtX process is the catalytic hydrogenation showed in Equation (10):

(10) $$\begin{array}{r} \text{C}{\text{O}}_{2}+{3\text{H}}_{2}⇌\text{ C}{\text{H}}_{3}\text{OH}+{\text{H}}_{2}\text{O }{\Delta \text{H}}_{298\text{K}}=-49.2\text{ kJ}/\text{mol} \end{array}$$

The reaction takes place at 250–300°C and 50–100 bars usually in the presence of CuO/ZnO/Al_2_O_3_ catalysts (Jadhav et al., 2014; Bellotti et al., 2017). In this process, hydrogen is produced by water electrolysis ideally using renewable energy, as presented in section Water Electrolysis, and then combined with CO_2_ waste streams to produce methanol, in a classical Power-to-Methanol process.

Recent process simulations and techno-economic studies have focused on the efficiency of Power-to-Methanol processes and on its comparison with the classical routes (Table 4). Rihko-Struckmann et al. (2010) investigated the idea of capturing the CO_2_ from power plants to produce energy storage media, such as H_2_ and methanol. They considered a H_2_ production from water electrolysis and the methanol production from a Power-to-Methanol technology at 220°C and 5 MPa. They achieved a high CO_2_ conversion of about 97%. However, they concluded that the exergetic efficiency of the system using hydrogen as storage medium was higher than the one including methanol. The main advantage of using methanol as chemical storage system would be, however, its simple and cost-efficient storage. Hank et al. (2018) evaluated the economic feasibility of a methanol plant with a methanol production capacity of 4–10 kt/y using CO_2_ and hydrogen as feedstock. The hydrogen used in the process was produced via a PEM water electrolyser with an electricity consumption of 4.76 kWh/m^3^ H_2_. They concluded that the feasibility of the process strongly depended on the costs of electricity and of H_2_ production, on the price of the CO_2_ (considering the carbon taxes) as well as on the dynamics of the methanol reactor including the necessity of H_2_ storage. They also concluded that locations with high availability and low cost production of renewable energy will play an important role on the development of Power-to-Methanol technologies in the next few years. Bellotti et al. (2017) compared the economic feasibility of three different plant sizes for methanol synthesis from hydrogen, produced by water electrolysis, and carbon dioxide, sequestrated from power plants. They concluded that in the case of large plant sizes with a methanol production around 50 kt/y considerable amounts of CO_2_ (71.6 kt/y) would be sequestrated. However, due to the high cost of the plant, oxygen selling would be mandatory to get economically feasible results. Koytsoumpa et al. (2018) showed that a large-scale methanol plant with a methanol production of 50–100 kt/y would require 1.01 MWhth per ton of CH_3_OH of thermal energy and 9.74 MWhe/t CH_3_OH of electric energy. The techno-economics are highly influenced by the final methanol price, the fuel and chemical market as well as the energy and fuel directives. Kourkoumpas et al. (2016) performed a techno-economic evaluation of the Power-to-Methanol concept using CO_2_ from lignite-fired power plants and H_2_ from water electrolysis. They concluded that the Power-to-Methanol concept is more competitive when large scale plants, low electricity, low CO_2_ costs and high operating time for both H_2_ and methanol plants are considered.

**Table 4:** Summary of some of the process simulations and techno-economic studies about Power-to-Methanol reported in the literature.

**T (°C)**	**P (bar)**	**CO_**2**_ conversion to MeOH (%)**	**Methanol production**	**Electricity consumption (kWh/kg MeOH)**	**H_**2**_ source**	**Electricity consumption (kWh/m^**3**^ H_**2**_)**	**References**
–	40	90	4–10 kton/y	–	Water electrolysis (PEM)	4.76	Hank et al., 2018
240	80	96	97 kg/h	–	Water electrolysis (PEM)	5.2	Bellotti et al., 2017
220	50	96.8	3.03 kmol/h	–	Water electrolysis	–	Rihko-Struckmann et al., 2010
–	–	–	50–100 kton/y	9.89	Water electrolysis (AEL)	4.4	Koytsoumpa et al., 2018

A few Power-to-Methanol plants are actually already in operation and projects aiming at building pilot and demonstration plants are currently being developed. The European Commission has launched the MefCO_2_ project aiming to demonstrate the economic feasibility of the Power-to-Methanol technology in a modular intermediate scale and to adapt it to varying plant sizes and gas composition. The technology could later be adapted to work with the existing biomass combustion and gasification system streams, for example, aiming in this case the production of electric/thermal energy instead of chemical synthesis^13^,^14^ (Koytsoumpa et al., 2018). Mitsui Chemicals built a pilot plant in 2008 for methanol production from CO_2_ and H_2_ obtained from water photolysis with a capacity of 100 tons of CH_3_OH per year. Their objective is to use the methanol produced as raw material to the synthesis of olefins and aromatics^15^ (Pérez-Fortes et al., 2016). Carbon Recycling International (CRI) has taken the technology one step further since the company developed an emission-to-liquid technology in which CO_2_ captured from flue gas of a geothermal power plant and H_2_ produced from water electrolysis using renewable energy are used to produce renewable methanol. The George Olah plant recycles 5.5 thousand tons of CO_2_ per year, releasing 90% less CO_2_ than the use of a comparable amount of energy from fossil fuels^16^.

### Methane

Methane (CH_4_) is one of the most important energy vectors of our society, being used to produce heat, electricity and value-added chemicals. Methane is mainly obtained through natural gas, a fossil fuel source with very low cost (≈ 3.13 USD/GJ—December 2018)^17^. If produced in a sustainable way, CH_4_, also called substitute natural gas (SNG), has also a great potential for reducing the GHG emissions, since it can be more readily used than renewable H_2_, for example. Indeed, SNG can be injected directly into the natural gas grid, benefiting of the existing natural gas facilities (Götz et al., 2016). Power-to-Methane (PtM) technology has shown to be a promising pathway for a sustainable production of methane, using CO_2_ and renewable energy to produce SNG. Among the different existing routes for PtM, catalytic CO_2_ hydrogenation (methanation) has been extensively investigated and demonstration plants are already in operation in different countries. CO_2_ electrochemical reduction route is still at lab-scale validation stage. However, the results obtained over the last few years have highlighted the promising aspects of this route.

#### CO_2_ Electrochemical Reduction

One of the possible ways to sustainably produce fuels (such as methane from CO_2_) is by an electrochemical reduction of CO_2_. Thermodynamically, CO_2_ can be electrochemically reduced to CH_4_ with a standard potential of +0.17 V vs. RHE (Peterson and Norskov, 2012). However, since hydrogen evolution reaction (HER) is thermodynamically possible at 0 V (vs. RHE), both reactions will be in competition at all negative potentials (Peterson and Norskov, 2012). The low CO_2_ solubility in water (0.03 M, 25°C and 1 atm) is also a remaining challenge. So, studies are still needed to improve CO_2_ solubility and to develop catalysts able to improve selectivity to methane and to reduce the high potentials required. Alternative approaches to CO_2_ electrochemical reduction, such as photo irradiation, use of ionic liquid electrolytes and use of biological microorganisms have also been treated elsewhere (Kondratenko et al., 2013; Machado et al., 2018) and are not the focus of this section.

Hori et al. (1994) pioneered experimental works on the comprehension of CO_2_ electrochemical reduction by heterogeneous catalysts. The authors performed CO_2_ reduction tests at 18.5°C in a 0.1 M KHCO_3_ medium using different metals, such as Cu, Au, Ag, Zn Pd, Cd, Ni, Pt, etc. They proved that methane could be obtained by CO_2_ electrocatalytic reduction on metal surfaces and that the choice of the metal of the electrode had an impact of the products selectivity. The only metal investigated that presented a relatively high Faradaic efficiency to CH_4_ (33.3%) was copper at −1.44 V vs. normal hydrogen electrode (NHE). Pd, Cd, and Ni also produced CH_4_ but with Faradaic efficiencies lower than 3%, mainly due to the HER occurring as side reaction. The good performance of copper was attributed to the fact that CO_2_ is intermediately reduced to CO, before being reduced to methane (and other hydrocarbons and alcohols). When other metals, such as Ni and Pt were used, the CO was adsorbed on the surface of the electrodes, preventing its further reduction into hydrocarbons and alcohols.

To the best of our knowledge, the best CH_4_ Faradaic efficiency obtained up to date on copper electrodes was obtained by Manthiram et al. (2014). The authors compared the performance of copper nanoparticles supported on glassy carbon (n-Cu/C) with classical copper foils. The electrochemical tests were performed in a two-compartment electrochemical flow cell separated by a Selemion membrane and in a 0.1 M NaHCO_3_ electrolyte. A Faradaic efficiency for CH_4_ of 76% was obtained using n-Cu/C (−1.35 V vs. RHE), which was significantly higher than the Faradaic efficiency of 44% obtained with the classical copper foil. The good performance of the n-Cu/C catalyst was attributed to the formation of nanoscale aggregates on the n-Cu/C upon polarization, exposing its catalytic sites to methanation. On the other hand, in copper foils highly connected networks of fused particles are formed upon polarization, leading to less exposed catalytic sites.

The electrolyte concentration as well as the local pH have also shown to have great influence on the selectivity of the CO_2_ electrochemical reduction. It has been proved that the methane formation depends on proton concentrations, being favored when acidic or neutral solutions are used. Thus, the effect of the electrolyte can be directly linked to the local pH. Varela et al. (2016) investigated the effect of the concentration of the electrolyte in controlling the selectivity of the CO_2_ electroreduction on copper. KHCO_3_ electrolyte solutions were used with concentrations varying between 0.05 and 0.2 M KHCO_3_. The highest concentrations of electrolyte favored the selectivity to methane. CH_4_ Faradaic efficiency of ≈ 70% at −1.43 V (vs. NHE) was obtained when a 0.2 M KHCO_3_ solution was used, due to its higher buffer capacity. The authors hypothesized that concentrated KHCO_3_ solutions had a higher buffer capacity and favored the electron/proton couple transfer, resulting in a higher methane (and H_2_) production. On the other hand, methane formation was less favored in diluted KHCO_3_ solution with low buffer capacity, due to the low concentration of protons close to electrode surface.

Issues related to the low CO_2_ solubility in water often leads to mass transfer limitations for large current densities. To overcome such problem, Hara et al. (1995) performed CO_2_ reduction using gas diffusion electrodes (GDE). Electrolysis experiments were carried out at 30 atm in a stainless steel autoclave using a 0.5 M KHCO_3_ aqueous electrolyte and a gas diffusion electrode containing Pt electrocatalysts. The Pt catalyst layer was directed toward the CO_2_ gas phase while the gas diffusion layer faced the aqueous electrolyte. Methane was produced with a Faradaic efficiency of 34.8% at a −1.92 V (vs. Ag/AgCl). However, when the order of the layers was inversed (Pt catalyst layer facing aqueous electrolyte), negligible amounts of methane were produced, mainly due to the contact between the water and the Pt catalyst, favoring the hydrogen evolution reaction.

Also aiming to improve CO_2_ solubility, Kaneco et al. (2002) investigated the electrochemical reduction of CO_2_ to methane at low temperatures in methanol, since it is a better solvent for CO_2_ as compared to water. Experiments were performed in a H-type cell with a Nafion 117-type ion exchange membrane as diaphragm using Cu electrodes. The authors reported a CH_4_ Faradaic efficiency of 58% at −2.0 V (vs. Ag QRE). The authors further investigated the use of different sodium salts (NaNO_3_, NaH_2_PO_4_, NaHCO_3_, NaCl, NaBr, NaI, NaF, Na_2_SO_4_, NaSCN, NaClO_4_, and CH_3_COONa) to further increase the CO_2_ solubility in methanol (Kaneco et al., 2006). The electrolysis tests were performed at 243 K using Cu electrodes. The authors reported that for all the sodium salts tested, CH_4_ Faradaic efficiencies ≥ 43.4% were obtained, 70.5% being the highest efficiency obtained using NaClO_4_. These results were attributed to the fact that the sodium salts depressed the hydrogen evolution reaction that competes with the CO_2_ reduction, thus increasing the methane formation.

#### CO_2_ Hydrogenation

Power-to-Methane (PtM) can also be achieved by combining water electrolysis for H_2_ production with methanation process in a two-step process. In the first step, H_2_ is produced using renewable energy, such as solar and wind power. In the second step, CO_2_ is hydrogenated in a methanation process to produce methane (Equation 11). Since the substitute natural gas (SNG) produced has high purity, it can be injected directly into the natural gas grid, stored or used in natural gas facilities (Götz et al., 2016), allowing this technology to connect the electrical and gas grids in a single system, procuring great flexibility to the balance of the grid (Bailera et al., 2017).

(11) $$\begin{array}{r} \text{C}{\text{O}}_{2}+{4\text{H}}_{2}⇌\text{C}{\text{H}}_{4}+{2\text{H}}_{2}\text{O }{\Delta \text{H}}_{298\text{K}}=-164.9\text{ kJ}/\text{mol } \end{array}$$

Methanation can be done either biologically or catalytically. However, the focus of this section will be on catalytic methanation, since it is (to the best of our knowledge) the most advanced technology. Catalytic methanation (Equation 11) is an exothermic reaction usually performed between 200 and 550°C and at pressures up to 100 bar. Due to its exothermicity, temperature control inside the reaction is an issue that can lead to thermodynamic limitations and catalyst deactivation by sintering (Götz et al., 2016). Therefore, different reactors types, such as fixed bed, fluidized bed, three-phase and structured reactors have been used to improve the efficiency of the process. Adiabatic fixed-bed reactor is the most commonly studied reactor type and is actually employed by companies such as Sasol, Linde, Haldor Topsoe etc., for the production of SNG from coal or naphtha (Schaaf et al., 2014; Götz et al., 2016). However, catalysts must be resistant against sintering due to the large temperature range they have to support because of the adiabatic mode (Götz et al., 2016). Usually, there are at least two fixed bed reactors connected in series for a good control of the reaction temperature, which is done by recirculating the reactor outlet gas stream and by intermediate gas cooling steps (Schaaf et al., 2014). Fluidized bed reactor is the second reactor type most commonly studied where the methanation reactions occur in a fluidized catalyst bed, improving heat and mass transfer during the reaction and thus the control of the process. Nevertheless, abrasion and entrainment of catalyst bed particles are some drawbacks of this type of reactor (Schaaf et al., 2014).

The catalyst has also a significant impact on the efficiency of the methanation process and Nickel-based catalysts are commonly used to this purpose due to their high activity and low cost (Ghaib and Ben-Fares, 2018). However, they impose limits to the methanation process, such as operating temperatures that must be kept between 200 and 550°C since potentially highly toxic nickel compounds can be formed at 200°C and catalyst deactivation by sintering and coking can occur above 550°C (Schaaf et al., 2014). So, studies have been carried out on catalyst development, aiming at synthetizing more performing and temperature-resistant catalysts. Bacariza et al. (2019) investigated the effect of the zeolite structure on the performances of Ni-based catalysts for CO_2_ methanation. Commercial available USY, BEA, ZSM-5 and MOR zeolites ion-exchanged with Na^+^ and Cs^+^ and impregnated with 15 wt.% Ni were tested at temperatures ranging from 250 to 450°C and GHSV of 43,000 h^−1^. The authors concluded that the performance of the catalysts was intimately related to the structure of the zeolites. USY zeolite showed the best catalytic performance with CO_2_ conversions of about 70% and CH_4_ selectivity close to 100%. The good catalytic performance was attributed to its weak interaction with water, which has an inhibitory role in the reaction, and to the high dispersion of the Ni particles over the catalyst surface. BEA zeolite led to the highest Ni particles dispersion and thus presented similar catalytic performances to the USY zeolite. Also, the catalytic performance of the BEA zeolite was improved when its hydrophobicity was increased by increasing its Si/Al ratio. Finally, ZSM-5 and MOR zeolites presented the lowest Ni dispersion, leading to lower methane selectivity around 60–70%.

Hydrotalcite materials have also been investigated due to the possibility of receiving high amounts of active phase while keeping high metal dispersion (Frontera et al., 2017), favoring CO_2_ conversion and preventing the catalyst deactivation. Abate et al. (2016) compared the performance of Ni-Al/hydrotacilte catalysts with a classical Ni/Al_2_O_3_ catalyst both doped with 75 wt.% Ni. The Ni-Al/hydrotacilte presented the best catalytic performance with a CO_2_ conversion of 85% and CH_4_ selectivity of about 85% at 275°C, while the Ni/Al_2_O_3_ commercial catalyst presented a CO_2_ conversion of around 80% and CH_4_ selectivity of about 75% at the same temperature. The difference in the catalytic performances was attributed to a higher nickel dispersion and a higher metal surface area of the Ni-Al/hydrotacilte catalyst. Similar results were obtained by He et al. (2014). The authors compared the performance of a Ni-Al/hydrotacilte catalyst with a Ni/Al_2_O_3_ catalyst both doped with 78 wt.% of Ni. Ni-Al/hydrotacilte catalyst presented a better catalytic performance with CO_2_ conversion of 82.5% and CH_4_ selectivity of 99.5% at 350°C, which was also attributed to a high nickel dispersion.

Noble metals have also been extensively investigated due to their good catalytic performance for CO_2_ methanation at low temperatures and good resistance to carbon formation (Frontera et al., 2017; Qin et al., 2017). Karelovic and Ruiz (2012) studied the performance of Rh/γ-Al_2_O_3_ catalyst with Rh content varying between 1 and 5 wt.% at a temperature range of 50–200°C. They reported that selectivity to methane was about 100% in all conditions investigated. They also reported that the turnover frequency (TOF) of methane formation decreased as the Rh dispersion decreased (increase in Rh content) in a temperature range of 135–165°C. However, the TOF was independent of the Rh dispersion in a higher temperature range (185–200°C). Swalus et al. (2012) investigated the effect of mechanically mixing Rh/γ-Al_2_O_3_ with Ni/activated carbon (AC) catalyst, a catalyst widely used for hydrogenation reactions. The objective was to verify the synergetic effect of the two catalysts in the CO_2_ methanation reaction. The reactions were performed at 125°C and 2 bar. The authors reported a higher methane production (9.5 μmolCH_4_/g_cat_) when both catalysts were used compared to when only Rh/γ-Al_2_O_3_ (6.8 μmolCH_4_/g_cat_) or Ni/AC (0 μmolCH_4_/g_cat_) was used. Since no new structures were formed when the catalysts were mixed, the synergy was attributed to the cooperation between the two catalysts during the reaction. Rh/γ-Al_2_O_3_ was highly efficient for adsorbing CO_2_ while Ni/AC was able to adsorb high quantities of H_2_. These two properties together improved the CO_2_ conversion and the CH_4_ formation. The authors suggested the hydrogen species would migrate from the surface of the Ni/AC toward the Rh/γ-Al_2_O_3_, reacting with the adsorbed CO_2_ to produce CH_4_. Basic promoters, such as K, Ba and CeO_2_ have also been studied to enhance the performance of the catalysts by modifying the surface basicity, the metal-support interaction as well as the metal dispersion. All these parameters would lead to a better activity, stability and resistance to extreme conditions of the catalysts (Ghaib and Ben-Fares, 2018).

As previously presented, there has been a lot of work on the different steps of the Power-to-Methane process, such as electrolysis and CO_2_ methanation. However, a lot of work still need to be done on the integration of these steps. Different countries, especially in Europe, driven by the need to increase the share of renewable energy started research projects to provide proof of concept to the Power-to-Methane technology. There are a few plants using this technology that are already in operation and others that are still been developed. A non-exhaustive list of these plants and projects is provided in Table 5.

**Table 5:** Summary of the PtM plants current in operation and of the PtM projects being developed.

**Plant/Company**	**Technology**	**Eff. (%)**	**CO_**2**_ conversion**	**CH_**4**_ production rate**	**Source of power**	**Power input (MW)**	**Electricity consumption (kWh/kg CH_**4**_)**	**H_**2**_ source**	**References**
Audi E-GAS/Audi	CO_2_ methanation	54	2.8 kt/y	1 kt/y (max:325 Nm^3^/h)	wind	6	13.85	AEL	Kondratenko et al., 2013; Bailera et al., 2017
ZSW 250-kWel	CO_2_ methanation	–	–	–	–	–	–	AEL	Schollenberger et al., 2018
Store&Go	CO_2_ methanation	–	–	–	–	1		AEL	^13^
HELMETH	CO_2_ methanation	>85	–	1.08–5.42 m^3^/h	–	–	–	SOEC	^14^ Ghaib and Ben-Fares, 2018

The largest Power-to-Methane facility in the world is the 6 MW Audi e-gas plant. In this plant, hydrogen is produced by alkaline electrolysers using wind power. The source of the CO_2_ is the biogas from the EWE Biogas GmbH & Co. KG biomethane plant. The methane production is limited to 1,000 t/y due to availability of the renewable energy. However, the maximum methane production capacity of the plant is 325 Nm^3^/h (Bailera et al., 2017).

ZSW launched in 2012 a Power-to-Methane demonstration plant composed by a 250-kWel alkaline high-pressure electrolyser, a CO_2_ methanation unit and a process control system^18^ (Ghaib and Ben-Fares, 2018). In this plant, two different reactor types (tube bundle and plate reactors) can be operated separately or in combination. Also, a membrane gas processing stage is used to enrich the methane in the product gas and to recycle the hydrogen-rich gas (Schildhauer and Biollaz, 2016).

The Store&Go project funded by the European Union developed a metallic honeycomb-like carrier-based reactor aiming at overcoming the issues related to removing and reutilizing the heat from the methanation reactor (Schollenberger et al., 2018). The proof of concept was done at laboratory scale. The technology was scaled up to a 1MW SNG plant, which started its operations in 2018^19^.

The HELMETH project, co-financed by the European Union and by the Fuel Cells and Hydrogen Joint Technology Initiative, aims at determining the conditions for an economic feasibility of the Power-to-Gas process and at demonstrating the technical feasibility with conversion efficiency >85%^20^. (Ghaib and Ben-Fares, 2018). The innovation of this project is the thermally integration of a high temperature electrolysis (SOEC) with methanation. The heat released during the methanation process will be used to vaporize the water that will be fed to the electrolyser, helping balancing the endothermal and exothermal process and allowing a better energy-efficiency storage of the renewable energy^14^.

Despite the key role that this technology might play in the future energy sector, further studies are still needed to validate the whole system as well as to overcome main drawbacks, such as low efficiency and high costs (Götz et al., 2016).

### Alkanes

The transportation sector is one the sectors having the largest GHG emissions in many countries. In Canada, for example, the transportation sector accounted for 25% (173 Mt CO_2eq_) of the total national emissions^21^, showing the critical need to displace the fossil fuels used in the sector to effectively reduce the GHG emissions of the country. Electric cars represent a promising alternative, however, these technologies are not yet optimized for long distance trips and are still unfit for heavy transportation. Biofuels have also been considered as a promising approach, though its use has raised a lot of questions regarding the amounts that can really be produced to significantly reduce the GES emissions from this sector (Schmidt et al., 2018). The challenge is even bigger when it comes to the aviation sector, since there is not yet a reliable substitute to the fossil-based jet fuel. The Power-to-Liquids (PtL) technology could represent a huge opportunity to produce a replacement fuel without the issues related to the biomass feedstock and that could actually reduce the emissions from the transportation sector, especially the aviation sector.

**Figure 4:**
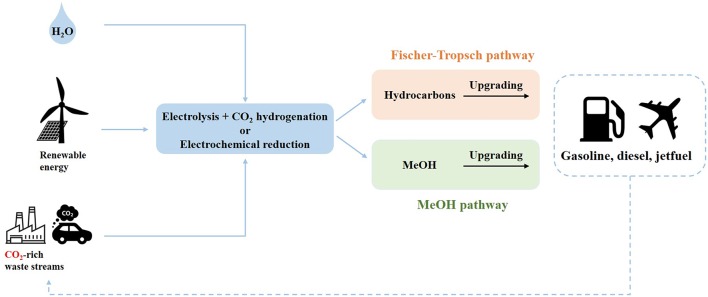
Power-to-Liquids (PtL) technology.

Fischer-Tropsch and methanol are the two main production pathways to produce alkanes through PtL technologies (Figure 4). To fit with the Fischer-Tropsch pathway, CO_2_ first is converted to CO via a reverse water-gas shift reaction (RWGS) and H_2_ is produced through water electrolysis (Schmidt et al., 2016). H_2_ and CO are then used in a classical Fischer-Tropsch synthesis to produce hydrocarbons that can be upgraded to fuels such as gasoline, diesel and jet fuel. There are several upgrading processes that are already widely employed for upgrading crude oil to jet fuel that could also be applied in the PtL, such as hydrocracking, isomerization and distillation (Schmidt et al., 2016). In the methanol pathway, H_2_ produced from water electrolysis, and CO_2_ (or CO) are used in the synthesis of methanol as an intermediate. Methanol can be then converted to fuels through further steps already used at industrial scale, such as DME synthesis, olefin synthesis, oligomerization, and hydrotreating (Schmidt et al., 2016).

To the best of our knowledge, there is no known reports relating the direct electrochemical reduction of CO_2_ into long chain hydrocarbons. Most of the CO_2_ hydrogenation studies focus on the synthesis of short-chain products, such as methane, methanol, formic acid etc., as presented in the previous sections. Nevertheless, recent studies reported the successful production of gasoline, diesel and jet fuel either via FT pathway or via MeOH pathway (Figure 4). The following sections will present a few recent studies on the production of these alkanes via CO_2_ hydrogenation.

#### Gasoline

Gasoline (C_5_-C_11_ hydrocarbons) is one of the most important transportation fuel used worldwide. Besides the classical production through petroleum refining, it can be synthesized via Fischer-Tropsch (direct route) or via methanol as intermediate (indirect route). This section will present the recent advances on gasoline production through these two pathways.

Wei et al. (2017) recently reported the production of gasoline via direct CO_2_ hydrogenation over a multifunctional Na–Fe_3_O_4_/HZSM-5 catalyst. Hydrogenation tests were performed in a fixed-bed reactor at 320°C and 3 MPa. The catalysts exhibited high selectivity to C_5_-C_11_ of 78%, very low selectivity to methane and CO (<10%) and high stability over 1,000 h of time on stream (TOS). The authors concluded that the Fe_3_O_4_ sites enabled the RWGS reaction while the zeolite sites favored the oligomerization/aromatization and isomerization. Moreover, the gasoline fraction was composed mainly by isoparaffins and aromatics, having a positive impact on the octane number. The authors further reported that the composition of C_5_-C_11_ could be tuned by using the zeolites with different topologies. HZSM-5 produced 61% of aromatics in the gasoline fraction while HMCM-22 produced mainly paraffins (≈46%). Despite the high selectivity of the catalysts, CO_2_ conversion reached only 22%.

Wang et al. (2016) investigated the production of C_5+_ isoalkanes, which can be used as gasoline additive, over a Fe-Zn/zeolite core-shell catalysts prepared by cladding method. Different zeolites (HZSM-5, Hbeta, HY) were investigated in order to verify the influence of their structure in the CO_2_ hydrogenation reaction at 340°C and 5 MPa for 2 h. Fe–Zn–Zr@HZSM-5–Hbeta double-zeolite shell catalyst with a HZSM-5/Hbeta ratio of 4 showed the highest isoalkanes/total hydrocarbons ratio (83.1%). These results were attributed to the synergistic effect of the HZSM-5 that promotes the production i-C_5+_, with Hbeta zeolites that favors the formation of i-C_4_. However, CO_2_ conversion was very low (around 14%). The authors also reported that the cladding method used for the preparation of the catalysts was very promising due to its simplicity and low cost, being suitable for use at large scale.

Gao et al. (2017) investigated the performance of a bifunctional catalyst containing partially reducible metal oxides (In_2_O_3_) and H-form Zeolite Socony Mobil-5 for the conversion of CO_2_ into liquid fuels using methanol as intermediate. The catalysts showed a high selectivity gasoline-range hydrocarbons (C_5+_) of 78.6% along with very low methane selectivity of 1% for over 150 h of TOS at 340°C and 3 MPa. The authors reported that the oxygen vacancies on the In_2_O_3_ activated the CO_2_ and the H_2_, producing methanol. Then, a C-C coupling occurs in the zeolite pores, converting methanol to C_5+_ hydrocarbons via a hydrocarbon-pool mechanism. As for all the studies presented up to date on the PtL for gasoline production, CO_2_ conversion was also very low around 13%.

#### Diesel

Diesel is the main transportation fuel used nowadays for heavy transportation. However, the literature related to CO_2_ hydrogenation into diesel is very scarce. Recently, Han et al. (2017) proposed a new path for direct CO_2_ conversion into liquid fuels with renewable hydrogen produced via solar water splitting. CO_2_ hydrogenation was performed at 300°C and 10 bar over a new Cu-Fe catalyst, which exhibited excellent catalytic performances with 65% selectivity to C_5+_ liquid hydrocarbons and only 2–3% methane selectivity. The authors reported that the main products of the reaction cover the gasoline (C_5_-C_11_) and diesel range (C_12_-C_21_), the product distribution being very similar to the one observed in CO-FT over iron-based catalysts. The performance of the catalyst was attributed to the swift reduction and selective carburization form of the Hagg iron carbide formed, which is actually the active phase for the production of long-chain hydrocarbons in the CO_2_ hydrogenation process.

One of the most significant studies is the CO_2_-to-diesel process developed by Audi in partnership with Sunfire, generating a carbon-neutral diesel fuel, called e-diesel. The process is performed in three main steps. In the first step, H_2_ is produced from high-temperature water electrolysis using renewable energy. Then, H_2_ reacts with CO_2_ (from a biogas facility) under high pressure and high temperature, producing long-chain hydrocarbons, called blue crude. In the final step, the blue crude is refined into e-diesel similarly to the fossil crude oil refining process^22^. The company started the production of e-diesel in 2015 and has produced more than three tones of blue crude up to date^23^.

Most of the data reported in the literature about CO_2_ hydrogenation into diesel-like fuels are actually related to the production of dimethyl ether (DME), which is considered a greener alternative to traditional diesel fuels due to its lower NO_x_ emissions, near-zero smoke, less carbon particulates and less engine noise (Wang et al., 2009; Álvarez et al., 2017; Li et al., 2018a). The MeOH pathway is the main route investigated, being used either in a two-step process in which MeOH is produced from CO_2_ hydrogenation in the first step (Equation 10) after what MeOH is dehydrated in a second step (Equation 12), or in a single-step process, in which both steps are done simultaneously in the same reactor (Wang et al., 2009; Li et al., 2018a).

(12) $$\begin{array}{r} {2\text{CH}}_{3}\text{OH}⇄\text{C}{\text{H}}_{3}\text{OC}{\text{H}}_{3}+{\text{H}}_{2}\text{O }{\Delta \text{H}}_{298\text{K}}=\;-23.4\text{ kJ}/\text{mol} \end{array}$$

Since CO_2_ conversion could be thermodynamically limited at low pressures if MeOH is not continuously removed from the reaction medium, its further conversion into DME in a single step process is preferred (Álvarez et al., 2017). So, studies for catalyst development for this process have focused on the synthesis of bifunctional catalysts capable of performing both reactions (Equations 10 and 12) in a single reactor. In general, catalysts used for each reaction separately are combined in a single bifunctional catalyst, which is synthesized by homogenous mixing of both catalysts, by sequential arrangement or even by homogenous mixing and grinding followed by pelletizing (Roy et al., 2018). It is widely accepted that a simple homogenous mixing results in more performing catalysts, since they maintain their initial properties (Roy et al., 2018). Copper and Zinc-based catalysts, for example, have been intensively investigated for the CO_2_ hydrogenation into methanol (see section CO_2_ Hydrogenation), first step of the process. ZnO is responsible for adsorbing CO_2_ while Cu adsorbs H_2_. The second step (MeOH dehydration) is favored by acid catalysts, such as alumina and zeolites (An et al., 2008). So, An et al. (2008) regrouped the functions of both catalysts in a bifunctional catalyst prepared by a physical mixture of CuO-ZnO-Al_2_O_3_-ZrO_2_ and HZSM-5 for the CO_2_ hydrogenation into DME in a single-step process. Reaction temperature and pressure varied between 483 and 543 K and 2 and 5 MPa, respectively. The best catalytic results were obtained at 543K and 5 MPa with CO_2_ conversion of about 27% and DME selectivity of 15.8%, proving that the authors could successfully synthesize DME from one-step CO_2_ hydrogenation. Similarly, Wang et al. (2009) investigated the performance of CuO-TiO_2_-ZrO_2_/HZSM-5 mixed oxides catalyst for producing DME from CO_2_ hydrogenation in a one-step process. The catalyst with a Ti/Zr ratio of 50/50 presented the best catalytic performance with DME selectivity of 47.5% (yield = 7.41%) and CO_2_ conversion of 15.6%. The results were attributed to the higher reducibility of this catalyst. Despite the advances on DME synthesis from CO_2_ hydrogenation over the last few years, low CO_2_ conversions and low DME selectivity and yield remain a bottleneck of this process.

#### Jet Fuel

Since there is no current alternative to fossil jet fuels, the Power-to-Liquids (PtL) technology could represent a huge opportunity to produce a replacement aviation fuel capable of effectively reducing the emissions from the aviation sector (Schmidt et al., 2018). The synthetic paraffinic kerosene produced from this process, for example, can be blended up to 50% to jet fuels (Schmidt et al., 2016).

There isn't yet a proof-of-concept of an integrated Power-to-Jetfuel technology. However, the individual steps have already high technological maturity level. Many big industrial actors have been developing this technology. Shell in partnership with other actors created the SOLAR-Jet consortium aiming to demonstrate a carbon-neutral pathway for producing jet fuel using solar energy^24^. The project explored the solar-thermochemical redox cycles between 2011 and 2015 and produced the world's first sample of solar thermochemical kerosene from H_2_O and CO_2_ at laboratory scale. The Sun-to-Liquid project created in 2016 succeeded the first project and aims to design, fabricate, and experimentally validate a large scale complete solar fuel production plant^25^.

Carbon Engineering's pilot Air to Fuels has successfully produced biocrude from CO_2_ and water in 2017. In this project, CO_2_ is captured from the air, purified and thermo-catalytically reacted with H_2_ produced from water electrolysis with renewable energy (solar PV), to produce the biocrude^26^. However, their major challenge is upgrading the biocrude into jet fuel. A commercial validation of the integrated technology is expected by 2021.

Carbon Recycling International's George Olah Renewable Methanol Plant in Svartsengi (Iceland) was completed in 2012 and produces 5 million liters of methanol per year. The plant uses hydro and geothermal energy for producing H_2_ from water electrolysis, which is then reacted with CO_2_ from flue gases to produce methanol. The methanol (vulcanol) produced can then be blended with gasoline for automobiles or used as intermediate in the production of fuels that could potentially be suitable as synthetic jet fuel (Schmidt et al., 2018).

## Conclusions

Power-to-X technology has gained increased attention since it tackles issues related to the production of carbon neutral fuels from CO_2_ and to the storage of renewable energy. The PtX technology includes two main steps: H_2_ production through water electrolysis using renewable energy and CO_2_ hydrogenation to chemicals and fuels. Alternatively, CO_2_ can be directly reduced into useful products via electrochemical reduction also using renewable energy.

Among the different methods for sustainably producing H_2_, water electrolysis is the main process investigated since it allows the production of high-purity hydrogen from renewable energy and water. AEL, PEM and SOEC are amongst the three most popular technologies for water electrolysis currently being investigated. Among the latter, AEL has the lower investments and maintenance costs and is the most mature, being already commercialized at MW scale. However, coupling it with variable renewable energy could be very challenging due to its long start-up preparation. On the contrary, SOEC is very promising when coupling with renewable energy is considered. Moreover, SOEC systems exhibits a great potential for coupling with exothermic processes as well as for CO_2_ and water co-electrolysis, but considerable progress is still required to get this technology to a next level. PEM exhibits strategic advantages related to high efficiency and short time response, being the most promising for PtX applications. The competitiveness of electrolysis in comparison to the other H_2_ production technologies should increase in the next few years due to the increase in the production of electrolysers as well as on the number of academic and industrial research projects related to this technology. Despite the important advances achieved in the past years, improvements in efficiency as well as cost reduction of electrolysis processes are still required in order to overcome technical and economic barriers to the successful commercialization of PtX.

The second step of the PtX process is the CO_2_ hydrogenation into chemicals and fuels either via a Fischer-Tropsch pathway or using methanol as intermediate (MeOH pathway). The CO_2_ hydrogenation process is more advanced than the other possible processes, such as CO_2_ electrochemical reduction, and it may benefit from the existing infrastructure of the classical Fischer-Tropsch process. The main researches on this process focus on the development of more efficient catalysts capable of withstanding the harsh reaction conditions and to selectively produce the desired products. Companies, such as Audi, Carbon Recycling International and ZSW have successfully built and operated PtX commercial plants for the production of methane and methanol.

Alternatively, CO_2_ can be directed converted into chemicals and fuels through the CO_2_ electrochemical reduction coupled with renewable energy. Contrarily to the CO_2_ hydrogenation process, this approach is still being developed at laboratory scale. The main drawbacks that hinders the scale up of this approach is the very low selectivity to the desired products, low efficiency as well as the high overpotentials required. Considerable technical and catalytic advances have yet to be achieved in order to get this technology to a larger scale.

Further development of the PtX technology will mainly require a decrease in the capital costs as well as an improvement of process efficiencies, especially electrolysis, leading to a reduction of the actual high production costs of fuels and chemicals produced through this technology. Validation of the different steps at large scale is also required. Despite the increase in the number of research projects in the area in partnership with industries, data related to the scale-up of the different steps of the technology is still missing. Finally, the integration of these steps to validate the whole PtX process is mandatory to help the technology reach higher technology readiness levels.

## Author Contributions

BRV and J-ML both contributed to defining the relevant topics and to the writing of the manuscript.

### Conflict of Interest Statement

The authors declare that the research was conducted in the absence of any commercial or financial relationships that could be construed as a potential conflict of interest.

## Footnotes

^1^https://treaties.un.org/pages/ViewDetails.aspx?src=TREATY&mtdsg_no=XXVII-7-d&chapter=27&lang=en

^2^https://www.cbc.ca/news/canada/where-canada-s-surplus-energy-goes-1.1109321

^3^http://www.betaengineering.com/high-voltage-industry-blog/transmitting-electricity-at-high-voltages

^4^https://www.britannica.com/topic/coal-utilization-122944/Gasification#ref623926

^5^https://www.audi.com.au/au/web/en/models/layer/technology/g-tron/power-to-gas-plant.html

^6^http://biocat-project.com/

^7^https://www.hydrogenics.com/2018/07/16/north-americas-first-multi-megawatt-power-to-gas-facility-begins-operations/

^8^https://industry.airliquide.ca/air-liquide-invests-worlds-largest-membrane-based-electrolyzer-develop-its-carbon-free-hydrogen

^9^https://blog.topsoe.com/how-can-electrolysis-of-water-help-to-produce-fuel-for-your-future-car

^10^https://www.fuelcellenergy.com/storage/

^11^https://www.toshiba-energy.com/en/hydrogen/rd/#rd02

^12^https://www.methanex.com/our-business/pricing

^13^http://www.mefco2.eu/mefco2.php,

^14^https://cordis.europa.eu/project/rcn/193453_en.html

^15^https://www.mitsuichem.com/en/release/2008/080825e.htm

^16^http://carbonrecycling.is/george-olah/

^17^https://www.nasdaq.com/markets/natural-gas.aspx)

^18^https://www.zsw-bw.de/en/research/renewable-fuels/topics/power-to-gas.html

^19^https://www.storeandgo.info/press-media/

^20^http://www.helmeth.eu/index.php/technologies/methanation-process

^21^https://www.canada.ca/en/environment-climate-change/services/environmental-indicators/greenhouse-gas-emissions.html

^22^https://www.audi-mediacenter.com/en/press-releases/fuel-of-the-future-research-facility-in-dresden-produces-first-batch-of-audi-e-diesel-352

^23^https://www.sunfire.de/en/company/news/detail/sunfire-produces-sustainable-crude-oil-alternative

^24^http://www.solar-jet.aero/page/about-solar-jet/objectives.php

^25^https://www.sun-to-liquid.eu/

^26^http://carbonengineering.com/about-a2f/
